# The traditional chinese medicine monomer *Ailanthone* improves the therapeutic efficacy of anti-PD-L1 in melanoma cells by targeting c-Jun

**DOI:** 10.1186/s13046-022-02559-z

**Published:** 2022-12-15

**Authors:** Pian Yu, Hui Wei, Kaixuan Li, Shiguo Zhu, Jie Li, Chao Chen, Detian Zhang, Yayun Li, Lei Zhu, Xiaoqing Yi, Nian Liu, Panpan Liu, Shuang Zhao, Xiang Chen, Cong Peng

**Affiliations:** 1 The Department of Dermatology, Xiangya Hospital, Central South University, Xiangya Road #87, Hunan 410000 Changsha, China; 2 National Clinical Research Center for Geriatric Disorders, Xiangya Hospital, 410000 Changsha, Hunan China; 3 Hunan Key Laboratory of Skin Cancer and Psoriasis, Xiangya Hospital, Xiangya Road #87, Hunan 410000 Changsha, China; 4 Hunan Engineering Research Center of Skin Health and Disease, Xiangya Hospital, 410000 Changsha, Hunan China; 5 Xiangya Clinical Research Center for Cancer Immunotherapy, Central South University, 410000 Changsha, Hunan China; 6 Laboratory of Integrative Medicine, School of Basic Medical Sciences, Shanghai University of Traditional Chinese Medicine, Shanghai, P. R. China

**Keywords:** Melanoma, *Ailanthone*, PD-L1, c-Jun, Treg, Tumor microenvironment

## Abstract

**Background:**

C-Jun, a critical component of AP-1, exerts essential functions in various tumors, including melanoma, and is believed to be a druggable target for cancer therapy. Unfortunately, no effective c-Jun inhibitors are currently approved for clinical use. The advent of immune checkpoint inhibitor (ICI) has brought a paradigm shift in melanoma therapy, but more than half of patients fail to exhibit clinical responses. The exploration of new combination therapies has become the current pursuit of melanoma treatment strategy. This study aims to screen out Chinese herbal monomers that can target c-Jun, explore the combined effect of c–Jun inhibitor and ICI, and further clarify the related molecular mechanism.

**Methods:**

We adopted a combinatorial screening strategy, including molecular docking, ligand-based online approaches and consensus quantitative structure-activity relationship (QSAR) model, to filter out c-Jun inhibitors from a traditional Chinese medicine (TCM) library. A mouse melanoma model was used to evaluate the therapeutic efficacy of monotherapy and combination therapy. Multicolor flow cytometry was employed to assess the tumor microenvironment (TME). Multiple in vitro assays were performed to verify down-streaming signaling pathway. CD4 + T-cell differentiation assay was applied to investigate Treg differentiation in vitro.

**Results:**

*Ailanthone* (AIL) was screened out as a c-Jun inhibitor, and inhibited melanoma cell growth by directly targeting c-Jun and promoting its degradation. Surprisingly, AIL also facilitated the therapeutic efficacy of anti-programmed death ligand-1 (PD-L1) in melanoma cells by reducing the infiltration of Tregs in TME. Additionally, AIL treatment inhibited c-Jun-induced PD-L1 expression and secretion. As a consequence, Treg differentiation was attenuated after treatment with AIL through the c-Jun/PD-L1 axis.

**Conclusion:**

Our findings identified AIL as a novel c-Jun inhibitor, and revealed its previously unrecognized anti-melanoma effects and the vital role in regulating TME by Treg suppression, which provides a novel combination therapeutic strategy of c-Jun inhibition by AIL with ICI.

**Graphical Abstract:**

AIL down-regulates c-Jun by reducing its stability, and inhibits the function of Tregs via AIL-c-Jun-PD-L1 pathway, ultimately suppressing melanoma progression and enhancing the efficacy of anti-PD-L1.

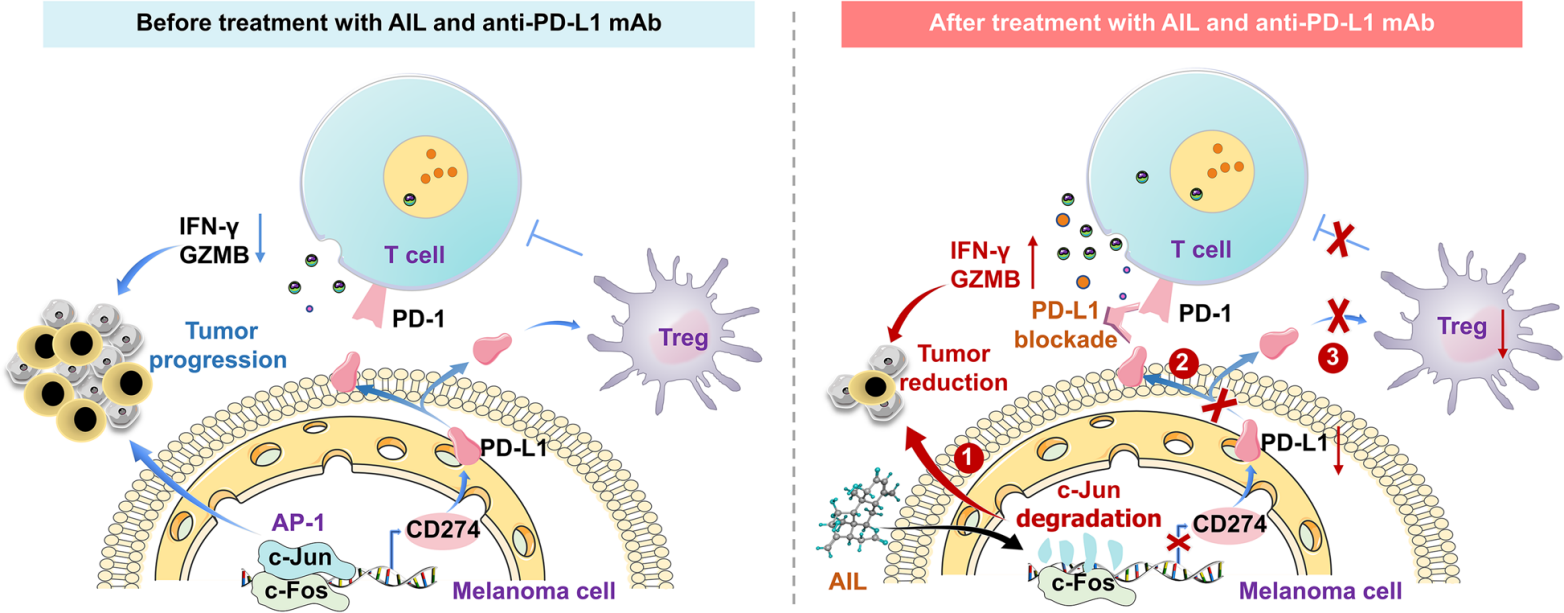

**Supplementary Information:**

The online version contains supplementary material available at 10.1186/s13046-022-02559-z.

## Background

Activator protein 1 (AP-1) is a pivotal transcription factor (TF) in tumorigenesis and exerts various biological functions, such as apoptosis, proliferation, migration, invasion and angiogenesis, in a variety of tumors [1–3]. The AP-1 protein consists of homodimers or heterodimers in the Jun proto-oncogene subfamily, including the c-Jun, JunB and Fos subfamily such as c-Fos and Fra1 [4]. The Jun family and Fos family are both shared on a basic leucine zipper (bZIP) domain, which comprises a DNA binding domain (DBD) and a leucine zipper (LZ) domain [5]. The LZ domain responds to form a homodimer of the Jun family member or a heterodimer of the Jun family and Fos family member, while the DBD domain is required for binding to DNA.

C-Jun is an important member of the AP-1 proteins, forming homodimers with Jun family members or heterodimers with Fos family, which has been well documented to be highly expressed in various tumors, including breast cancer, lung cancer and melanoma [6–8]. AP-1/c-Jun have emerged as actively pursued therapeutic targets worldwide, with potentially high therapeutic indexes [9]. Currently, the strategy for targeting c-Jun includes blocking the DBD domain bound to DNA or disrupting dimerization between c-Jun and other partners. MLN44 is an inhibitor that blocks c-Jun from associating with DNA and has exhibited antitumor activity in some tumors [10–12]. However, it is accompanied by off-target and side effects [13, 14]. T-5224 is another inhibitor that breaks the dimerization of c-Jun and c-Fos association with DNA based on a small peptide targeting the c-Jun and c-Fos DBD domains for arthritis therapy [15]. Unfortunately, phase II clinical trial results indicated that its effectiveness is low [9].

Cutaneous melanoma, derived from transformed melanocytes, is a mortal skin tumor, which is characterized by no response to chemotherapy or radiotherapy [16, 17]. The advent of immune checkpoint inhibitor (ICI), including programmed cell death protein 1 (PD-1) and its ligand 1 (PD-L1), has brought a paradigm shift in melanoma therapy [18], but more than half of patients fail to exhibit clinical responses [19]. The low response rate or resistance to anti-PD-1/PD-L1 therapy is partially due to immunosuppressive cells, including myeloid-derived suppressor cells (MDSCs) and regulatory T cells (Tregs), which suppress the function of effector cells [20, 21]. Tregs are a subtype of CD4(+) expressing T cells, featuring Forkhead box protein 3 (Foxp3) TF expression, which exerts a major suppressor for antitumor immune activity through different mechanisms, including mediating the expression of cytokines such as IL-10 and TGF-beta, suppressing antigen presentation and promoting cytolysis of effector cells [22].

PD-L1, also known as CD274, binds to PD-1, leading to blockade of immune cell activity, including CD8^+^ T cells, and maintaining immune homeostasis [23, 24]. Evidence has shown that PD-L1 has an essential role in Treg differentiation and maintaining Treg function. PD-L1 facilitated differentiation from naive CD4 + T cells to Foxp3 + Tregs [25, 26]. Moreover, PD-L1 inhibits AKT-mTOR signaling pathway, thereby increasing the expression of Foxp3 and enhancing the immunosuppressive ability of Tregs [25].

In this study, we found that *Ailanthone* (AIL) was a potential inhibitor of c-Jun by a ligand-based QSAR model combined with a structure-based docking approach from a traditional Chinese medicine (TCM) library, which exhibited remarkable antitumor activity against melanoma cells. More importantly, our findings showed that AIL significantly enhanced the efficiency of anti-PD-L1 therapy through suppression of Tregs differentiation by inhibiting the c-Jun-PD-L1 axis in melanoma cells, indicating that AIL combined with anti-PD-L1 therapy is a potential promising strategy for melanoma treatment.

## Materials and methods

### Cell culture, transfection and lentiviral infection

The human melanoma cell lines SK-MEL-5, SK-MEL-28, A375 and WM35, the mouse malignant melanoma cell lines B16-F10 and YUMM1.7, the immortalized human epidermal melanocyte cell line PIG1, and the murine epidermal cell line JB6 were purchased from American Type Culture Collection (ATCC). SK-MEL-5, SK-MEL-28, A375, WM35 and JB6 cells were cultured in Dulbecco’s modified Eagle’s medium (DMEM)/high glucose medium supplemented with 10% fetal bovine serum, PIG1 cells were cultured in Opti-MEM I Reduced Serum Medium supplemented with 10% fetal bovine serum, and YUMM1.7 cells were grown in Dulbecco’s Modified Eagle Medium/Nutrient Mixture F-12 (DMEM/F-12, 1:1) supplemented with 10% fetal bovine serum (FBS, Gibco, USA). Cells were cultured at 37 °C in a 5% CO_2_ humidified atmosphere.

### Reagents and antibodies

AIL was purchased from Nanjing DASF Biotechnology. CHX was purchased from Sigma (USA). MG-132 was purchased from Selleck (USA). An antibody against PD-L1 was purchased from Cell Signaling Technologies (CST, USA) and Abcam (USA). Anti-c-Jun was purchased from Santa Cruz Biotechnology (USA). Anti-GAPDH was purchased from Proteintech (USA).

### Transfection

For plasmid transfection, c-Jun overexpression was accomplished with a recombinant lentiviral vector GV358 (GeneChem, China), and the empty vector was used as a control. The lentiviruses were produced by GeneChem (China). In brief, cells in the exponential phase of growth were plated in 6-well plates at 1 × 10^5^ cells per well and grown for 24 h. LV-c-Jun or empty vector was seeded at an MOI of 20. After puromycin selection, the c-Jun-overexpressing cells (referred to as c-Jun-OE) and control cells (referred to as Ctrl-OE) were harvested.

### Cell cytotoxicity assay

Cells were seeded in 96-well plates and incubated for 24 h at 37℃. Next, the melanoma cells were treated with PBS or various concentrations of AIL for 0, 24, 48, or 72 h. Cell viability was detected with a Cell Counting Kit 8 (CCK-8; Selleck, USA) according to the manufacturer’s instructions. Fluorescence was measured with an emission wavelength of 450 nm at the end of incubation.

### Western blot

Cells were lysed with RIPA buffer (Beyotime, China) supplemented with a protease inhibitor cocktail (Selleck, USA). The protein concentration was measured by a BCA assay kit (Beyotime, China). Proteins were separated by 10% SDS-PAGE gels, transferred to polyvinylidene fluoride (PVDF) membranes (Millipore, USA), and visualized by western blotting using specific antibodies. The blots were captured by a gel image analysis system.

### Quantitative QRT-PCR analysis

Total RNA was extracted with TRIpure Reagent (Bio Teke, China), and cDNA was synthesized with HiScript Q RT SuperMix for reverse transcription PCR (Vazyme, China). GAPDH was used to normalize mRNA expression.

### Luciferase reporter gene assays

Cells were transfected with PD-L1-Luc and SV-40RenillaLuc for 24 h. Then, cell lysates were harvested and analyzed for firefly and Renilla luciferase activities with a dual luciferase assay kit (Promega, USA) according to the manufacturer’s protocol.

### Chromatin immunoprecipitation (ChIP) assay

The ChIP assay was performed using EZ-ChIP (Merck Millipore, USA) following the manufacturer’s instructions.

### Enzyme-linked immunosorbent assay (ELISA)

For the detection of PD-L1 in AIL-treated melanoma cell culture supernatants, a PD-L1 ELISA kit (Jianglai, China) was used to quantify the secretion of PD-L1 following the manufacturer’s protocol.

### Protein half-life assay

To examine the effect of AIL on c-Jun protein stability, melanoma cells were first treated with AIL for 24 h and then treated with cycloheximide (CHX; 200 mg/mL; Sigma, USA) for 0, 1, 2 and 4 h or MG-132 (30 µmol/L; Selleck, USA) for 8 h. We harvested cell lysates at the indicated time points for western blot.

### Pull-down assay

0.03 g of CNBr activated sepharose 4B (Sangon Biotech, China) was dissolved in 30 mL of 1 mM HCL, turned over and mixed, centrifuged at 2000 rpm for 2 min, and the supernatant was discarded. AIL was dissolved with coupling buffer, and the prepared CNBr activated sepharose 4B was added to the reaction system and spun overnight at 4 °C. The next day, the reaction system was centrifuged at 4000 rpm for 2 min, the supernatant was discarded, the precipitate was resuspended with 5ml coupling buffer, the precipitate was turned over and cleaned for 5 min, and the supernatant was discarded after centrifugation at 4000 rpm for 2 min. The precipitate was resuspended by 5ml of 0.1 M Tris-HCl (pH 8.0) buffer and flipped overnight at 4 °C. On the third day, the reaction system was centrifuged at 4000 rpm for 2 min, the supernatant was discarded, the precipitate was resuspended in 10 mL of 0.1 M acetic buffer (pH 4.0), the precipitate was turned over and cleaned for 5 min, and the supernatant was discarded after centrifugation at 4000 rpm for 2 min. The precipitate was resuspended in 10 mL of 0.1 M Tris-HCl + 0.5 M NaCl buffer (pH 8.0), washed by turning over for 5 min, and the supernatant was discarded after centrifugation at 4000 rpm for 2 min. When the prepared beads needed to be temporarily stored, 10 mL PBS + 1 µL 10% sodium azide solution was added and stored in a refrigerator at 4 °C. The cell lysates (SK-MEL-5, SK-MEL-28, B16-F10 and YUMM1.7) were incubated with AIL-linked Sepharose 4B beads (or Sepharose 4B alone as a control) in reaction buffer. After incubation with gentle rocking overnight at 4 °C, the beads were washed three times with wash buffer, and the proteins that bound to the beads were analyzed by western blot using c-Jun antibody.

### CD4 + T-cell differentiation in vitro

Naive CD4 + T cells were prepared using a CD4 + CD62L + T Cell Isolation Kit (mouse, Miltenyi Biotec, Germany). Next, the cells were transferred to a plate pretreated with CD3e monoclonal antibody (5 µg/mL, Invitrogen, USA) at 37 °C for 4 h, and then cultured under the stimulation of Purified NA/LE Hamster Anti-Mouse CD28 (2 µg/mL, BD Biosciences, USA) and recombinant mouse TGF-beta1 (5 ng/mL, R&D Systems, USA) in RPMI-1640 complete medium (Gibco) for 3 days. Cells were immediately collected for flow cytometry analysis.

### Xenograft tumor model

The animal experiments were approved by the Ethics Committee of Xiangya Hospital (Central South University) and strictly followed the “3R” principle of experimental animals (Ethics code: 201,803,363). B16-F10 cells were collected, washed with PBS three times, and resuspended in cold serum-free medium. B16-F10 melanoma cells (5 × 10^5^ cells, 100 µL RPMI-1640 medium) were injected into the right flank of 6- to 8-week-old female C57BL/6 mice (Shanghai SLAC Laboratory Animal). When the tumors were visible, the tumor-bearing mice were randomly grouped for intraperitoneal injection of AIL, anti-PD-L1 mAb (Bio X Cell, USA), IgG isotype control (IgG2a) (Bio X Cell, USA), or PBS (vehicle control) for 9–11 days. Tumor diameters were measured with a digital caliper every other day, and tumor volume was calculated according to the formula V = (length × width^2^)/2. The tumors were immediately collected for flow cytometry analysis.

### Dataset

To achieve comprehensive and diverse bioactivity data, we collected c-Jun data from the ChEMBL and BindingDB databases [27, 28]. The selection of c-Jun bioactivity data was refined with the following criteria: (1) removing compounds without explicit description for c-Jun and explicit molecular structures and retaining only compounds with IC50 and Ki values; (2) if there were two or more entries for a molecule, the arithmetic mean value of these values was adopted to reduce the random error. To maintain the balance of data used for modeling, we set 1000 mM as the cut-off activity value to split the dataset. The screening molecules used in this study were obtained from TCM monomers from the MCE compound library, consisting of 1616 compounds. The structures of these compounds were built and compiled by MOE (Molecular Operating Environment software, version 2016, Chemical Computing Group, Montreal, QC, Canada).

### Molecular docking

Molecular docking, a structure-based screening technique used in the drug design process, was employed to find the potential candidates and the interactions between hit molecules and proteins. The detailed methods are described in the article of Wei et al. [29].

### Consensus SAR modelling

The experimentally determined quantitative activity data for each target protein were collected from the BindingDB database. The methods referred to the article of Wei et al. [29].

### Ligand-based prediction approaches

To quickly narrow the potential targets of AIL, 4 commonly used ligand-based online prediction tools, SEA, PPB2, SwissTargetPrediction and HitPickV2, were applied as the strategy for target acquisition of AIL. The prediction approaches were performed as Wei et al. described [29].

### ADMET evaluation

The failure of many drug candidates in clinical trials may be due to poor absorption, distribution, metabolism, excretion, and toxicity (ADMET) properties. Here, we evaluated 8 ADMET properties for 6 hit compounds, including octanol/water partition coefficient (logP), human intestinal absorption (HIA), 30% bioavailability (F), blood brain barrier penetration (BBB), half-life (T 1/2), hERG blockers (hERG), human hepatotoxicity (H-HT), and ames mutagenicity (Ames).

### Data analysis

Data were analyzed by unpaired two-tailed Student’s t-test. All experiments were performed at least three times. Differences between groups were considered statistically significant at *p* < 0.05. GraphPad Prism software (version 8.0) was used for analysis.

## Results

### AIL identified from TCM monomers is a novel potential inhibitor targeting c-Jun

**Fig. 1:**
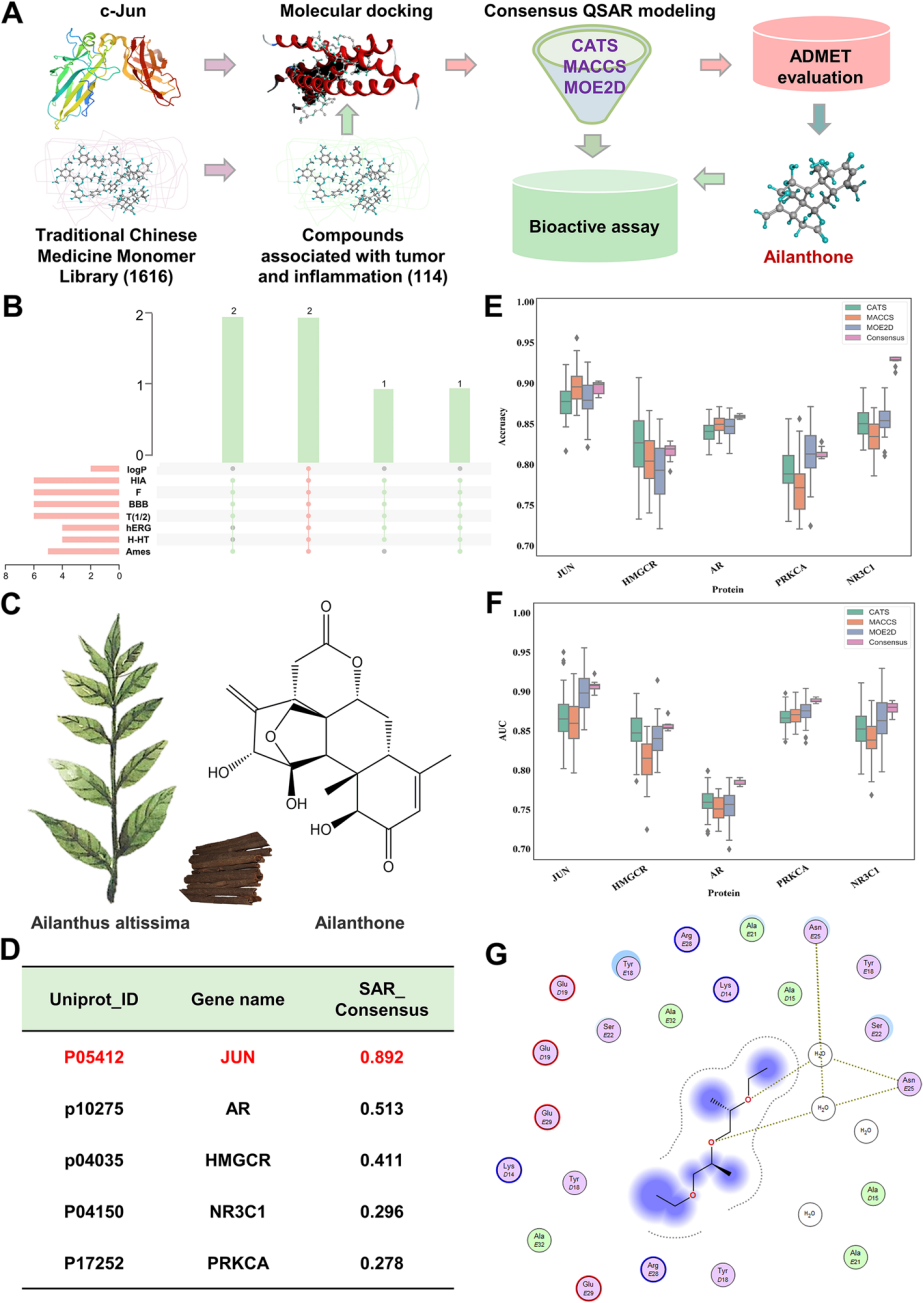
A virtual screening strategy identified c-Jun inhibitors. **A** Schematic representation of the virtual screening strategy. **B** An Upset plot of ADMET property analysis for the hits. **C** Molecular structure of AIL. **D** Potential targets of AIL predicted by our proposed strategy. **E** Accuracy scores of consensus QSAR models and single SAR models based on three descriptors. **F** AUC scores of consensus QSAR models and single SAR models based on three descriptors. **G** The optimal structural conformation of c-Jun and the ligand AIL

To develop c-Jun inhibitors, we focused on TCM monomers, the treasure trove of bioactive compounds, and screened out antitumor drug candidates with anti-inflammatory effects due to the rapid development of immunotherapy in the treatment of melanoma. 114 compounds (Additional file 1: Table S1) relevant to both tumor and inflammation were selected from 1,616 TCM monomers according to the literature, and then, we processed 114 compounds and c-Jun by MOE software (2016 version) as well as conducted molecular docking in induced fit mode. Finally, the 25 top compounds were initially screened out as potential inhibitors targeting c-Jun (Additional file 2: Fig. S1A). To obtain reliable c-Jun inhibitors, a ligand-based QSAR integration model was employed to further screen for the activity of inhibitors. Then, we collected a dataset of known binding small molecules of c-Jun from ChEMBL and BindingDB. Previous study proved that the consensus QSAR model constructed by CATS, MACCS and MOE2D, which performance is significantly better than a single model [30]. Therefore, we also adopted random forest (RF) to build a consensus QSAR model based on 5-fold cross-validation. Our results showed high prediction accuracy of the consensus QSAR model (Additional file 1: Table S2). With a value of 0.5 as the threshold, 6 compounds with predictive values greater than 0.5 were screened for further verification (Additional file 1: Table S3). Furthermore, the pharmacokinetics and toxicity properties of 6 potential inhibitors were calculated through ADMETlab (Additional file 1: Table S4). Among those candidates, *Britannin* (BRT) and AIL were finally identified as candidate inhibitors with high lipid solubility and water solubility, high bioavailability, low cardiotoxicity, and low hepatotoxicity (Additional file 1: Table S4 and Fig. 1 A-B).

AIL is the main active constituent extracted from the bark of Ailanthus altissima (Fig. 1C). To further validate that AIL was a targeted inhibitor of c-Jun, we performed a combinatorial target prediction strategy for the stepwise screening of the potential targets of AIL (Additional file 2: Fig. S2A). First, four ligand-based prediction platforms, including Swiss TargetPrediction, ChEMBL, Similarity Ensemble Approach (SEA) and PPB2, were employed to narrow the target space of the query molecule AIL [31], and we obtained 26 targets in total. Subsequently, we found that these targets were closely related to cancer and inflammation according to the top enriched KEGG pathways (Additional file 2: Fig. S2B), suggesting that the functions of preliminary screening targets were consistent with the phenotypic effects of AIL. To acquire reliable prediction, a Venn diagram was then plotted to observe the overlay of targets predicted by at least three prediction tools (Additional file 2: Fig. S2C). Finally, 5 molecules, including proto-oncogene c-Jun (JUN), androgen receptor (AR), 3-hydroxy-3-methylglutaryl-coenzyme A reductase (HMGCR), glucocorticoid receptor (NR3C1) and protein kinase C alpha type (PRKCA) (Fig. 1D), were identified as potential targets of AIL.

We also applied a consensus QSAR model to further screen the key targets of AIL. The evaluation metrics accuracy (ACC) and area under the curve (AUC) were all above 0.7 (Fig. 1E-F), indicating the good predictive ability of the QSAR models. The prediction results based on the consensus QSAR model were outputted as probability values and are listed in Fig. 1D. Here, with a probability value of 0.5 as the default cut-off, the proto-oncogene c-Jun and AR were selected as potential targets. A previous study reported that AIL downregulated AR protein levels [32]. Interestingly, our prediction showed that the c-Jun score was higher than the AR score (Fig. 1D), suggesting that our prediction was relatively reliable and that c-Jun was most likely to be the target of AIL. To further validate our hypothesis, molecular docking between c-Jun and AIL was performed to observe their binding. As shown in Fig. 1G, the main binding force is hydrogen binding; therefore, we confirmed the interaction between AIL and c-Jun.

### **AIL suppresses melanoma progression*in vitro*and*in vivo***

**Fig. 2:**
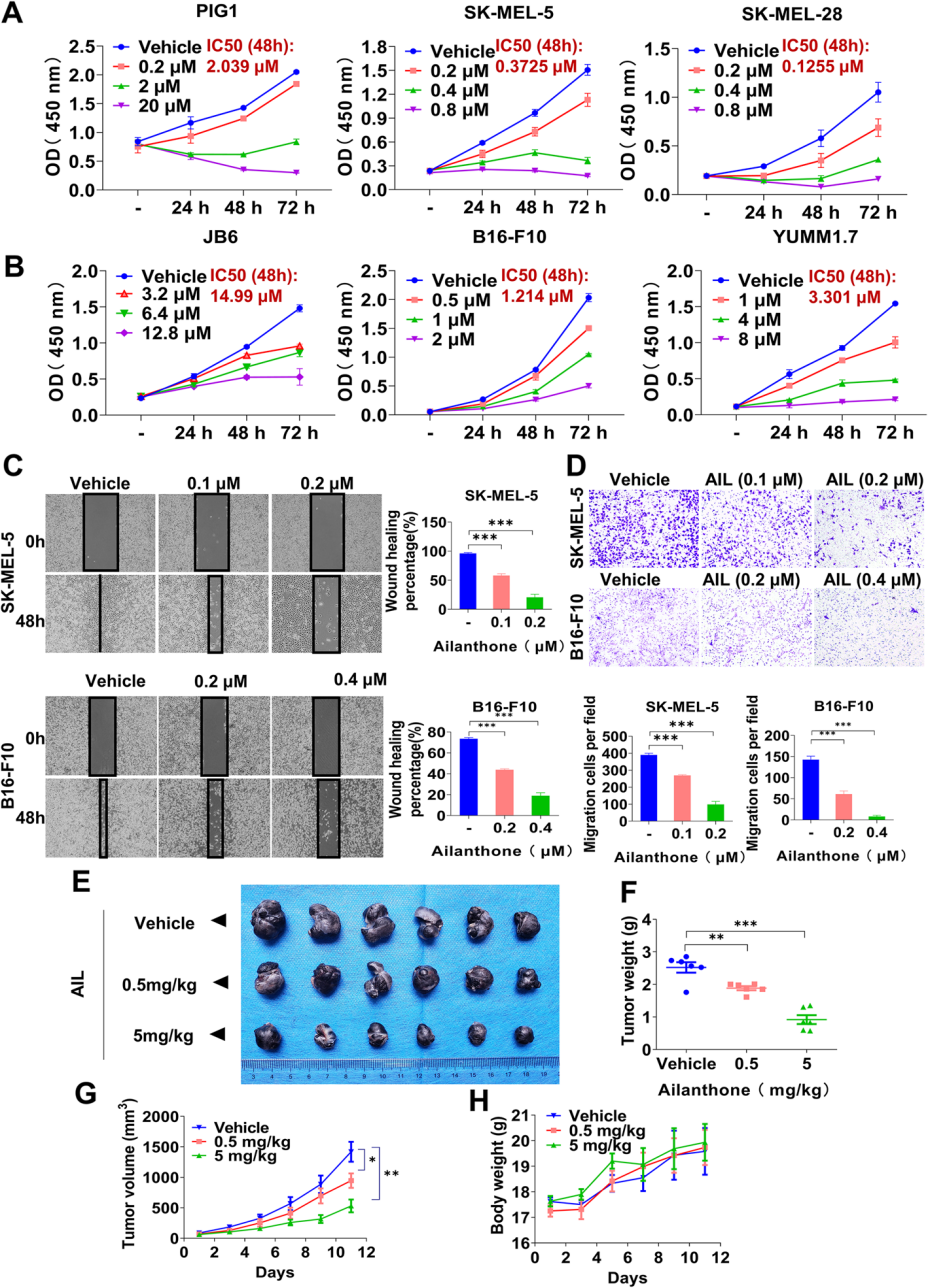
AIL inhibited melanoma progression in vitro and in vivo. **A** PIG1, SK-MEL-5 and SK-MEL-28 cells were treated with the indicated concentrations of AIL for 0, 24, 48 and 72 h. Cell viability was determined with the CCK-8 reagent. The average IC_50_ values for AIL in PIG1, SK-MEL-5 and SK-MEL-28 cells are shown. **B** JB6, B16-F10 and YUMM1.7 cells were treated with the indicated concentrations of AIL for 0, 24, 48 and 72 h. Cell viability was determined with the CCK-8 reagent. The average IC_50_ values for AIL in JB6, B16-F10 and YUMM1.7 cells are shown. **C** Wound healing assay in SK-MEL-5 and B16-F10 cells treated with AIL. Data are shown as the mean ± SEM, *n* = 3; *p < 0.05, **p < 0.01 and ***p < 0.001. **D** Transwell invasion assay in SK-MEL-5 and B16-F10 cells treated with AIL. Data were shown as the mean ± SEM of three independent experiments. Data are shown as the mean ± SEM, *n* = 3; *p < 0.05, **p < 0.01 and ***p < 0.001. **E**-**H** C57BL/6 mice were implanted with 5 × 10^5^ B16-F10 cells and received PBS or AIL (0.5 and 5 mg/kg). (E) Photographs of tumor samples isolated from C57BL/6 mice treated with PBS or AIL. **F** Tumor weight. **G** Volume changes in the tumor. **H** The body weight curves of the mice measured every two days. Data are shown as the mean ± SEM, *n* = 6; *p < 0.05, **p < 0.01 and ***p < 0.001

To study the role of AIL in melanoma, we examined the viability of the normal epidermal melanocytes (PIG1), the mouse epidermal JB6 cells, the human melanoma cell lines SK-MEL-5 and SK-MEL-28, and the mouse malignant melanoma cell lines B16-F10 and YUMM1.7 treated with AIL by CCK-8 assay. As shown in Fig. 2 A and Additional file 2: Fig. S3A, AIL inhibited cell proliferation with IC_50_ values (48 h) of 2.039 µmol/L in PIG1 cells, but inhibited cell proliferation with IC_50_ values (48 h) of 0.3725, 0.1255, 0.2245 and 0.6663 µmol/L in SK-MEL-5, SK-MEL-28, A-375 and WM35 cells, respectively. In addition, AIL inhibition of IC_50_ values (48 h) was calculated to be 14.99 µmol/L in JB6 cells, but inhibited cell proliferation with IC_50_ values (48 h) of 1.214 and 3.301 µmol/L in B16-F10 and YUMM1.7 cells, respectively (Fig. 2B), indicating that melanoma cells are more sensitive to AIL treatment than the normal cells. We next explored the effects of AIL on metastasis and invasion of melanoma cells by wound healing assay and transwell invasion assay. As shown in Fig. 2C-D and Additional file 2: Fig. S3B-C, AIL significantly inhibited the metastasis and invasion of the human and mouse melanoma cell lines. To further validate the antitumor effects of AIL, we inoculated B16-F10 mouse melanoma cells into C57BL/6 mice for an in vivo study. As shown in Fig. 2E-G, AIL treatment significantly reduced tumor volume and weight compared with the control group, and both 0.5 and 5 mg/kg AIL application did not cause a significant difference in mouse body weight among tumor-bearing mice (Fig. 2 H). Together, these results suggest that AIL serves as a promising therapeutic candidate in the treatment of melanoma.

### AIL binds to c-Jun and induces its degradation

**Fig. 3:**
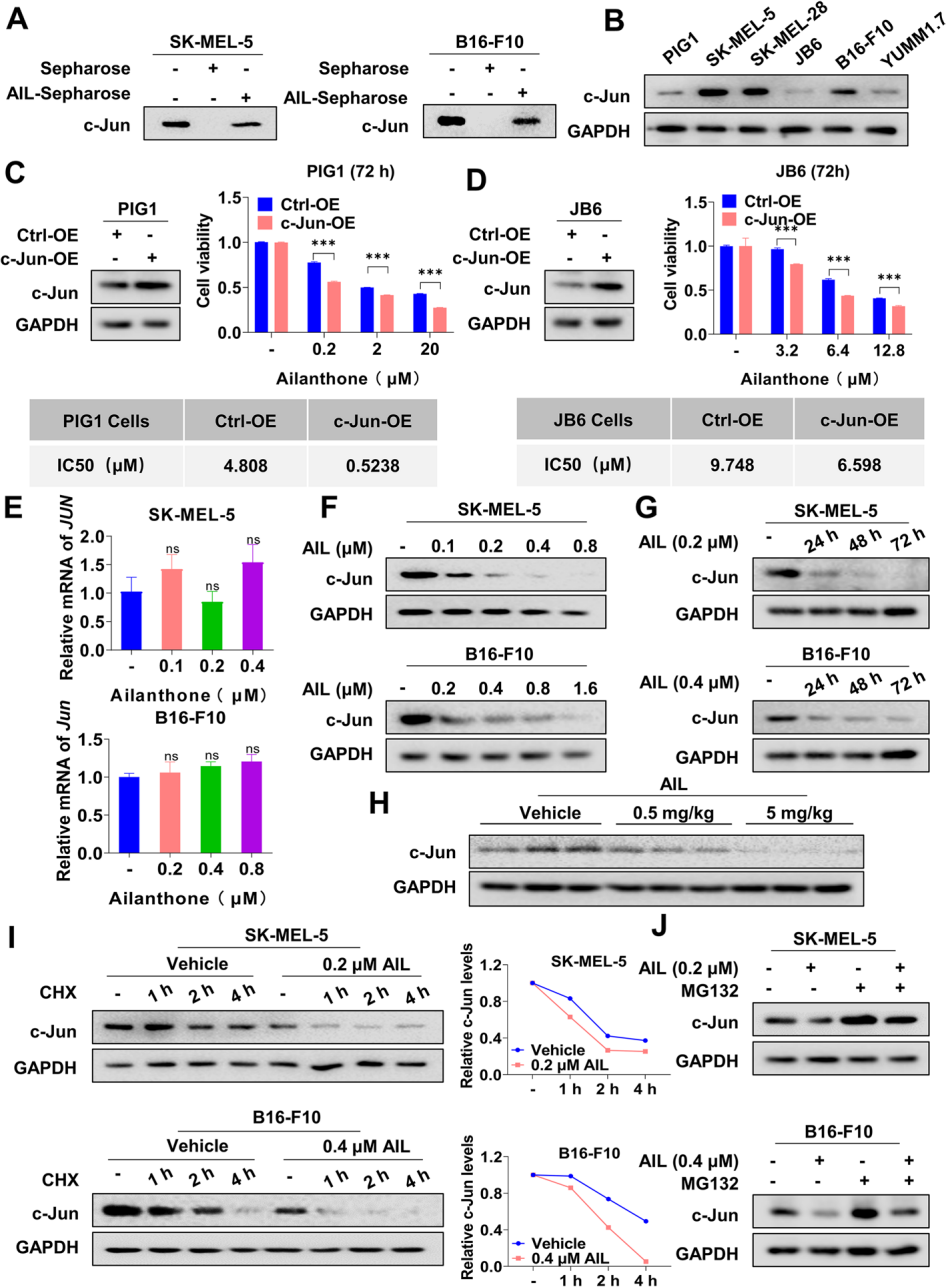
AIL directly interacted with c-Jun and promoted its degradation. **A** SK-MEL-5 and B16-F10 cell lysates were detected by western blot with anti-c-Jun antibody for the pull-down assay. **B** Differential expression levels of c-Jun in PIG1, SK-MEL-5, SK-MEL-28, JB6, B16-F10 and YUMM1.7 cells. The c-Jun protein level in PIG1, SK-MEL-5, SK-MEL-28, JB6, B16-F10 and YUMM1.7 cells was measured by western blot. GAPDH was used as the loading control. **C**-**D** PIG1 and JB6 cells were transfected with c-Jun overexpression or negative control vector, and c-Jun expression was analyzed by western blot. GAPDH served as the loading control. Cells were then treated with various concentrations of AIL for 72 h, and cell viability was examined by CCK-8 assay. **E** SK-MEL-5 and B16-F10 cells were treated with the indicated concentrations of AIL for 24 h. QRT-PCR was performed to analyze c-Jun mRNA levels. **F** SK-MEL-5 and B16-F10 cells were treated with the indicated concentrations of AIL for 24 h. Western blot was applied to detect c-Jun expression. GAPDH was used as a loading control. **G** SK-MEL-5 and B16-F10 cells were treated with AIL for different lengths of time. Western blot was applied to detect the c-Jun protein level. GAPDH was used as a loading control. **H** Western blot analysis of the expression of c-Jun in tumor samples with the indicated concentrations of AIL treatment. GAPDH was used as a loading control. **I** SK-MEL-5 and B16-F10 cells were treated with cycloheximide (CHX, 200 µg/mL) with or without AIL for the indicated time. The c-Jun protein level was measured by western blot. GAPDH was used as a loading control. **J **SK-MEL-5 and B16-F10 cells were treated with AIL with or without MG-132 (30 µmol/L), and c-Jun protein levels were measured by western blot. GAPDH was used as a loading control. Data are shown as the mean ± SD, *n* = 3; *ns*, not significant; *p < 0.05, **p < 0.01 and ***p < 0.001

Next, to verify the direct interaction between AIL and c-Jun in melanoma cells, we collected SK-MEL-5, SK-MEL-28, B16-F10 and YUMM1.7 cell lysates and incubated them with AIL-Sepharose 4B beads. The results showed that c-Jun bound to the AIL-Sepharose 4B beads complex but not the Sepharose 4B beads alone (Fig. 3A and Additional file 2: Fig. S4A). To further test whether c-Jun was a target of AIL, we detected c-Jun expression in PIG1, SK-MEL-5, SK-MEL-28, JB6, B16-F10 and YUMM1.7 cells. Compared with non-malignant cells, c-Jun was highly expressed in melanoma cells (Fig. 3B and Additional file 2: Fig. S5A). As expected, after transfecting c-Jun into PIG1 and JB6 cells, cell viability was enhanced (Additional file 2: Fig. S4B and Fig. S5B-C); however, these cells were more sensitive to AIL treatment than the control cells (Fig. 3C-D). To investigate the role of AIL in c-Jun, we detected the c-Jun mRNA and protein levels after AIL treatment in melanoma cells. As shown in Fig. 3E-G, Additional file 2: Fig. S4C-E and Fig. S5D-E, AIL had no significant effect on c-Jun transcription (Fig. 3E and Additional file 2: Fig. S4C) but reduced c-Jun protein expression in a dose-dependent and time-dependent manner in melanoma cells (Fig. 3F-G, Additional file 2: Fig. S4D-E and Fig. S5D-E). We also observed that AIL treatment resulted in a significant downregulation of c-Jun in tumor tissues compared with the control (Fig. 3H and Additional file 2: Fig. S5F). Since c-Jun is a highly unstable protein because of polyubiquitination [33], we wondered whether c-Jun was regulated by the proteasome pathway in AIL-treated melanoma cells. Intuitively, the half-life of c-Jun was shortened after AIL treatment (Fig. 3I and Additional file 2: Fig. S4F), and we also observed that MG-132, a proteasome inhibitor, could rescue the downregulation of c-Jun in melanoma cells after AIL treatment (Fig. 3J, Additional file 2: Fig. S4G and Fig. S5G). Thus, we provide evidence here that AIL targets c-Jun by reducing its protein stability.

### AIL improves the therapeutic efficacy of PD-L1 blockade by inhibiting the infiltration of Tregs

**Fig. 4:**
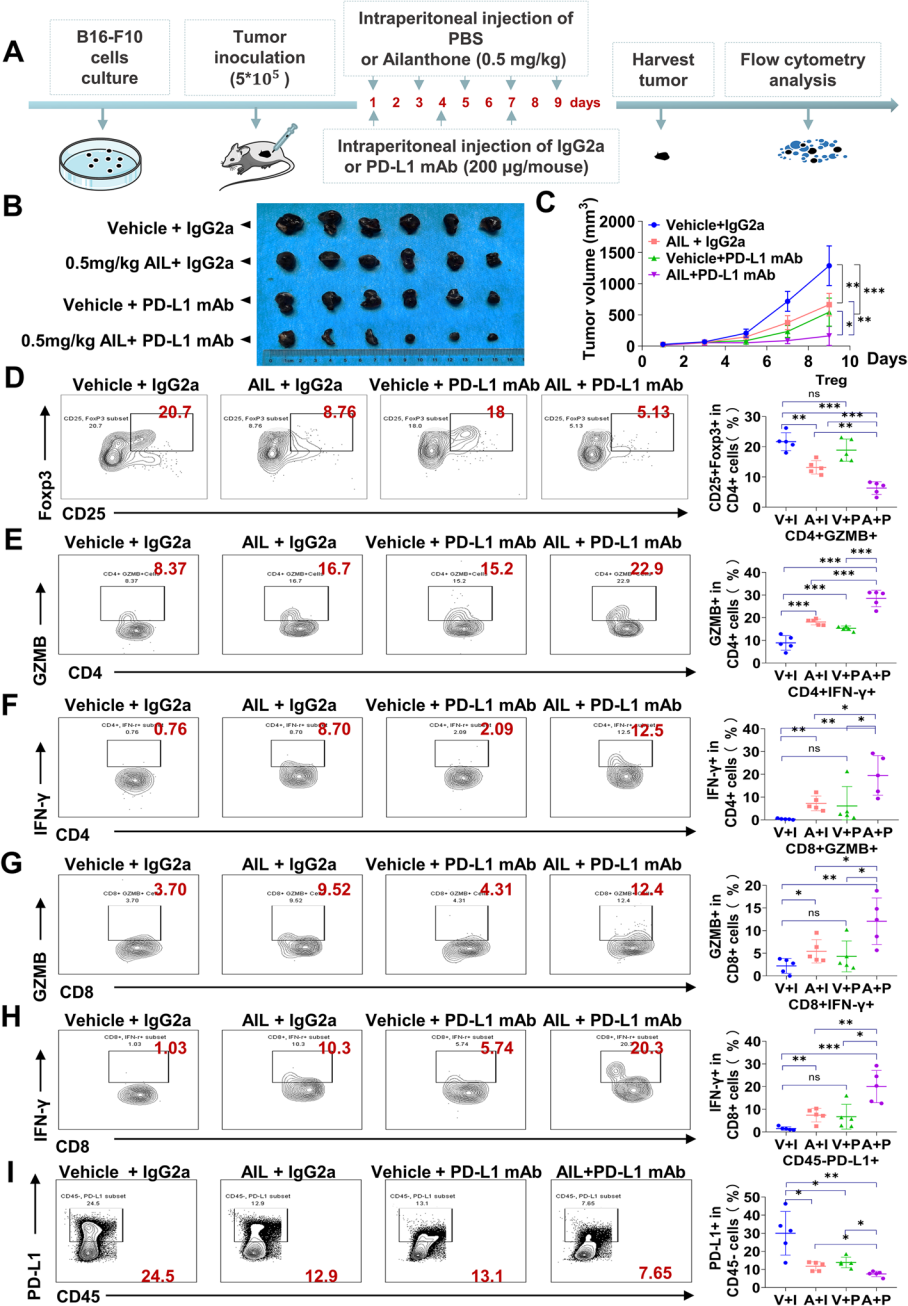
Improved antitumor effect of anti-PD-L1 mAb and AIL cotreatment in B16-F10 tumor-bearing C57BL/6 mice. **A-C** C57BL/6 mice were implanted with 5 × 10^5^ B16-F10 cells and received PBS, AIL, IgG isotype control (IgG2a) or PD-L1 mAb treatment (*n* = 6). **A** Schematic of the treatment plan. **B** Photographs of tumor samples isolated from C57BL/6 mice receiving the indicated treatments. **C** Tumor growth curves for tumors. **D** Representative flow cytometric plots and quantification of CD25 + FOXP3 + Tregs in CD4 + cells after the indicated treatments. **E** Representative flow cytometric plots and quantification of CD4 + GZMB + in CD4 + T cells after the indicated treatments. **F** Representative flow cytometric plots and quantification of CD4 + IFNγ + in CD4 + T cells after the indicated treatments. **G** Representative flow cytometric plots and quantification of CD8 + GZMB + in CD8 + T cells after the indicated treatments. **H** Representative flow cytometric plots and quantification of CD8 + IFNγ + in CD8 + T cells after the indicated treatments. **I** Representative flow cytometric plots and quantification of PD-L1 + in CD45- cells after the indicated treatments. Data are shown as the mean ± SEM, *n* = 5; *ns*, not significant; *p < 0.05, **p < 0.01 and ***p < 0.001

Combination therapy is a promising strategy to improve the efficacy of anti-PD-L1 mAb. To validate the effect of AIL treatment on immunotherapy, we utilized an anti-PD-L1 mAb to treat immune-competent mice inoculated with B16-F10 melanoma cells. We treated B16-F10 tumor-bearing mice with PBS plus IgG isotype CTRL (IgG2a), AIL plus IgG2a, PBS plus anti-PD-L1 mAb or AIL plus anti-PD-L1 mAb. Anti-PD-L1 treatment was performed every 3 days, and AIL treatment was performed every 2 days (Fig. 4 A). We captured tumors on the ninth day, and tumor growth was measured every 2 days (Fig. 4A). In the B16-F10 tumor-bearing mice, AIL or anti-PD-L1 mAb treatment alone both reduced mouse tumor burden (Fig. 4B). More importantly, cotreatment with AIL and anti-PD-L1 mAb further decreased xenograft tumor volume and weight (Fig. 4 C and Additional file 2: Fig. S6A). We observed that AIL, anti-PD-L1 mAb and combination treatments of AIL and anti-PD-L1 mAb had no significant difference compared with the CTRL group in terms of mouse body weight (Additional file 2: Fig. S6B). At the end of the treatment, tumor samples were harvested for further flow cytometry analysis. Interestingly, combined treatment with AIL and anti-PD-L1 mAb resulted in a significantly lower number of Tregs than each treatment alone (Fig. 4D). We also found that both AIL and anti-PD-L1 mAb increased the number of CD4 + T cells and CD8 + T cells, but the effect of combination therapy on CD4 + T cells and CD8 + T cells was not different from that of AIL or anti-PD-L1 mAb treatment alone (Additional file 2: Fig. S6C). Furthermore, we investigated the production of IFN-γ and granzyme B (GZMB) by CD4 + T cells and CD8 + T cells. As shown in Fig. 4E-H, the populations of IFN-γ + CD8 + T cells, GZMB + CD8 + T cells, IFN-γ + CD4 + T cells and GZMB + CD4 + T cells were significantly increased in both the AIL group and the anti-PD-L1 mAb group and synergistically improved by the combination treatment. We also detected the change in macrophages (F4/80 + CD11b+) and the percentage of Gr-1 + CD11b + MDSCs in CD45 + cells. We noticed an increase in the M1 (F4/80 + CD11b + MHC class II+) macrophage population in both the AIL treatment group and the anti-PD-L1 mAb group (Additional file 2: Fig. S6D). Additionally, AIL and anti-PD-L1 mAb treatment substantially reduced the infiltration of M2 (F4/80 + CD11b + CD206+) macrophages and MDSCs (Additional file 2: Fig. S6E and F). However, when AIL and anti-PD-L1 mAb treatment were combined, changes in M1 macrophages, M2 macrophages and MDSCs were no more significant than when AIL or PD-L1 was treated alone (Additional file 2: Fig. S6D-F). Compared to the control group, CD45-PD-L1 + cells showed a significant decrease upon AIL or anti-PD-L1 mAb treatment, which was further suppressed by combination treatment with AIL and anti-PD-L1 mAb (Fig. 4I), indicating that PD-L1 expression was distinctly inhibited in tumors. However, there were no significant differences detected in the populations of NK1.1 + cells, NK1.1 + PD-L1 + cells, CD4 + CTLA4 + cells, and CD8 + CTLA4 + cells (Additional file 2: Fig. S6G-J), suggesting that the immune effects of AIL and the anti-PD-L1 mAb were independent of these cells. Taken together, these results support the concept that AIL and anti-PD-L1 mAb treatment substantially reduce the Tregs and improve the cytotoxicity of T cells.

### AIL inhibits PD-L1 transcriptional expression by regulating c-Jun

**Fig. 5:**
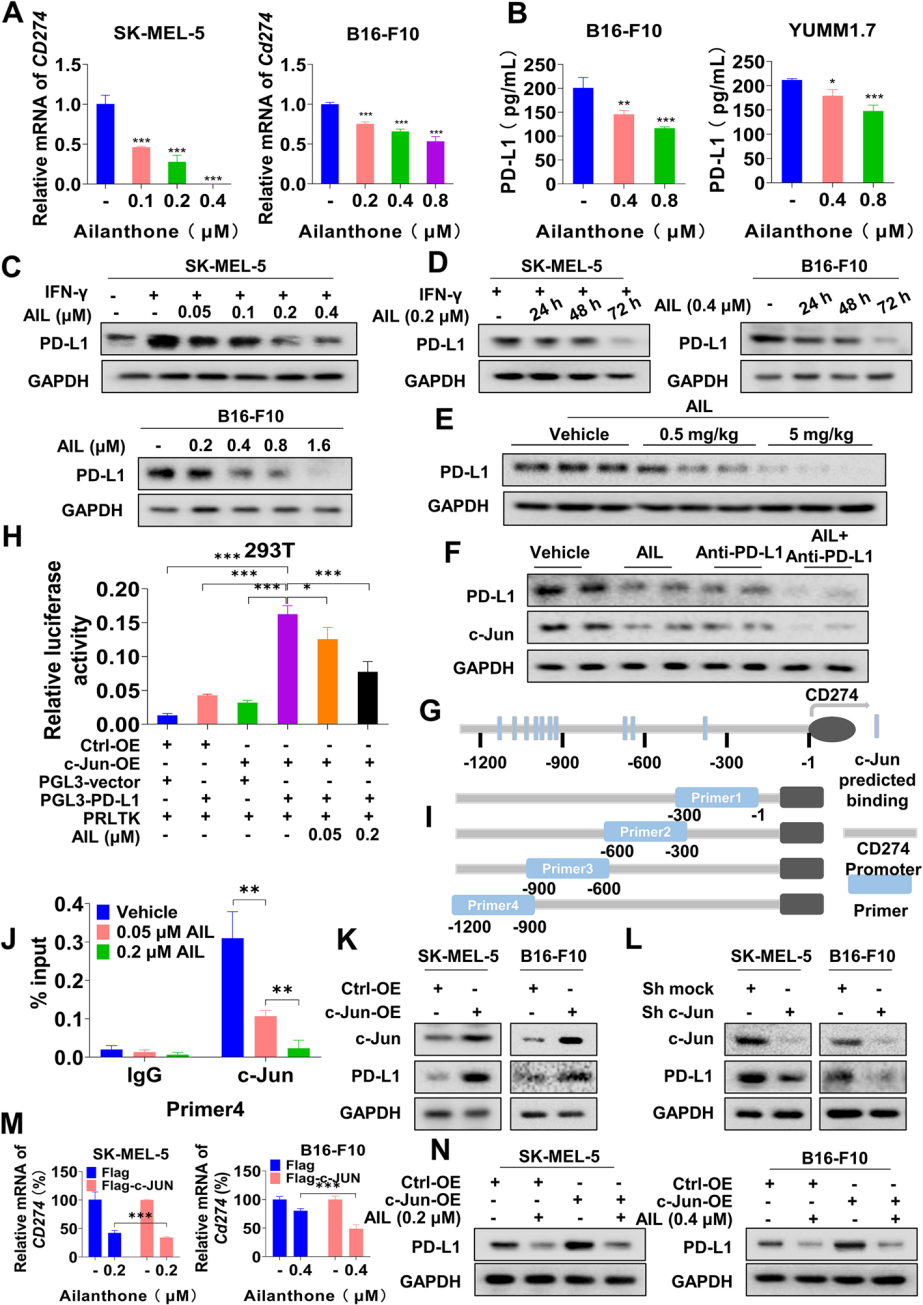
AIL suppressed PD-L1 transcriptional expression through c-Jun. **A** SK-MEL-5 and B16-F10 cells were treated with the indicated concentrations of AIL for 24 h. QRT-PCR was performed to analyze PD-L1 mRNA levels. **B** SK-MEL-5 and B16-F10 cells were treated with the indicated concentrations of AIL for 24 h, and the level of PD-L1 in the supernatant was determined by ELISA. **C** SK-MEL-5 and B16-F10 cells were treated with the indicated concentrations of AIL for 24 h. Western blot was applied to detect PD-L1 expression. GAPDH was used as a loading control. **D** SK-MEL-5 and B16-F10 cells were treated with AIL for different lengths of time. Western blot was applied to detect the PD-L1 protein level. GAPDH was used as a loading control. **E** Western blot analysis of the expression of PD-L1 in tumor samples with the indicated concentrations of AIL treatment. GAPDH was used as a loading control. **F** Western blot analysis of the expression of c-Jun and PD-L1 in tumor samples with anti-PD-L1 mAb and AIL cotreatment. GAPDH was used as a loading control. **G** c-Jun binding sites predicted on the website. **H** PD-L1 transcriptional activity in c-Jun plasmid-transfected 293T cells detected by dual luciferase reporter assays. **I** Designed primers for ChIP experiments. **J** ChIP–qPCR assays for c-Jun binding to the PD-L1 promoter sites in SK-MEL-28 cells with or without AIL treatment. **K** c-Jun and PD-L1 expression in SK-MEL-5 and B16-F10 cells overexpressing vector or c-Jun was detected by western blot in SK-MEL-5 and B16-F10 cells. **L** c-Jun and PD-L1 expression in SK-MEL-5 and B16-F10 cells with c-Jun knockdown was detected by western blot. **M** Relative mRNA levels of PD-L1 in SK-MEL-5 and B16-F10 cells overexpressing vector or c-Jun upon AIL treatment were analyzed by qRT–PCR. **N** PD-L1 expression in SK-MEL-5 and B16-F10 cells overexpressing vector or c-Jun upon AIL treatment was detected by western blot. Data are shown as the mean ± SD, *n* = 3; *p < 0.05, **p < 0.01 and ***p < 0.001

Previous results showed a reduction in PD-L1 expression after treatment with AIL (Fig. 4I). To identify the effect underlying AIL-mediated regulation of tumor PD-L1 levels, we detected the mRNA level of PD-L1 after AIL treatment in different melanoma cell lines. As shown in Fig. 5A and Additional file 2: Fig. S7A, AIL decreased PD-L1 mRNA levels in a dose-dependent manner. We also found that AIL significantly inhibited PD-L1 levels in the supernatant (Fig. 5B). Furthermore, western blot analysis showed that the expression of PD-L1 decreased in a dose- and time-dependent manner in cells treated with AIL (Fig. 5C-D, Additional file 2: Fig. S7B-C and Fig. S8A-B). We also observed that AIL treatment resulted in a significant downregulation of PD-L1 in tumor tissues compared with the control (Fig. 5E and Additional file 2: Fig. S8C). Interestingly, compared to the control group, c-Jun and PD-L1 protein levels showed a significant decrease upon AIL or anti-PD-L1 mAb treatment, which was further suppressed by combination treatment with AIL and anti-PD-L1 mAb in tumor tissues (Fig. 5F and Additional file 2: Fig. S8D). Previous studies have shown that c-Jun, the target of AIL, is a common TF mediating the transcription of many tumor-relevant genes, such as cyclin D1, p53, and INK4A [34–36]. We thus speculated that c-Jun was also a potential regulator of PD-L1 in melanoma cell lines. To verify our hypothesis, we identified the potential sequences in the promoter region of PD-L1 recognized by c-Jun (-1,200 bp~-1 bp) through the PROMO database (Fig. 5G) and constructed a PD-L1 luciferase reporter gene. The results of the luciferase assay showed that overexpression of c-Jun significantly enhanced PD-L1 promoter activity, which could be blocked by AIL treatment (Fig. 5H). In addition, we performed a ChIP and confirmed that c-Jun could directly bind to the promoter of PD-L1 and transcriptionally regulate PD-L1 mRNA levels, but AIL treatment inhibited their interaction (Fig. 5I-J and Additional file 2: Fig. S7D). To determine the downregulation of PD-L1 expression under AIL treatment through c-Jun inhibition, we overexpressed c-Jun in SK-MEL-5, SK-MEL-28, B16-F10 and YUMM1.7 melanoma cell lines (Fig. 5K, Additional file 2: Fig. S7E and Fig. S8E). To investigate that AIL inhibited PD-L1 expression via downregulating c-Jun, we knocked down the expression of c-Jun. Our results showed that suppression of c-Jun can reduce PD-L1 expression (Fig. 5L and Additional file 2: Fig. S8F). We observed that overexpression of c-Jun significantly increased the PD-L1 mRNA and protein levels compared to the control group (Fig. 5M, Additional file 2: Fig. S7F-I and Fig. S8G). In addition, overexpression of c-Jun partially rescued the downregulation of PD-L1 caused by AIL (Additional file 2: Fig. S7F-I). However, overexpression of c-Jun actually resulted in more significant inhibition of PD-L1 by AIL (Fig. 5N, Additional file 2: Fig. S7G-H and Fig. S8G). Taken together, our results support the hypothesis that AIL treatment reduces PD-L1 expression via the AIL-c-Jun-PD-L1 axis.

### AIL attenuates Treg differentiation through the c-Jun/PD-L1 axis

**Fig. 6:**
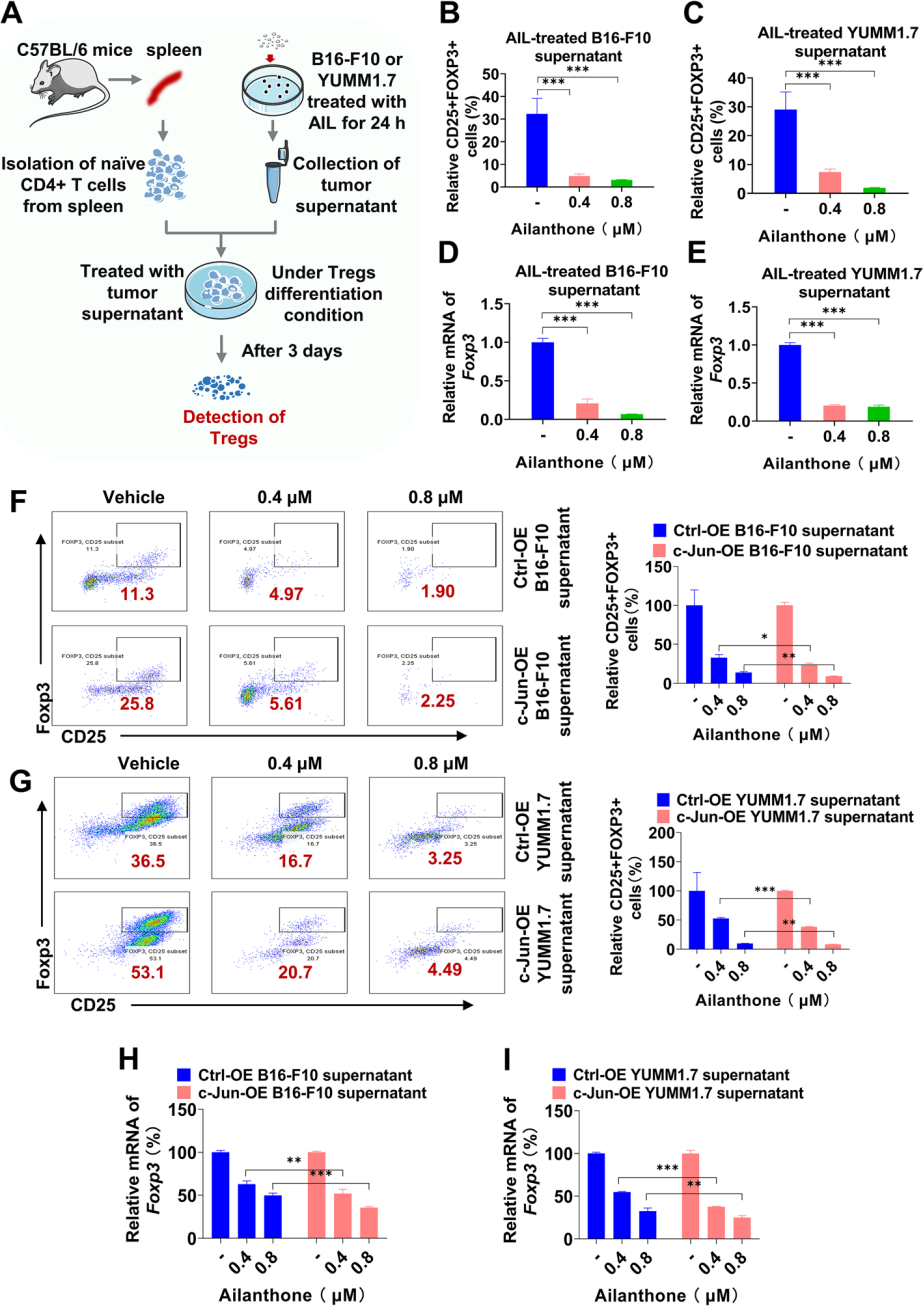
AIL blocked Treg differentiation through targeting c-Jun. **A-E** Naive CD4 + T cells were isolated from the spleen of C57BL/6 mice, seeded at a density of 1 × 10^6^ cells per well in 24-well plates, and finally cultured with AIL-treated B16-F10 supernatant or AIL-treated YUMM1.7 supernatant under Treg-prone conditions. After 3 days, CD25 + Foxp3 + cells were detected by flow cytometry, and Foxp3 mRNA levels were determined by qRT–PCR. (A) Schematic of the treatment plan. **B-C **Relative quantification of Tregs differentiated from naive CD4 + T cells. **D-E** Foxp3 mRNA level. **F-I** Naive CD4 + T cells were isolated from the spleen of C57BL/6 mice, seeded at a density of 1 × 10^6^ cells per well in 24-well plates, and finally cultured with AIL-treated vector/c-Jun-overexpressing B16-F10 supernatant or AIL-treated vector/c-Jun-overexpressing YUMM1.7 supernatant under Treg-prone conditions. After 3 days, CD25 + Foxp3 + cells were detected by flow cytometry, and Foxp3 mRNA levels were determined by qRT–PCR. **F-G** Representative flow cytometric plots and relative quantification of Tregs differentiated from naive CD4 + T cells. **H-I** Foxp3 mRNA level. Data are shown as the mean ± SD, *n* = 3; *p < 0.05, ** *p* < 0.01 and *** *p* < 0.001

In the TME, Tregs contribute to tumor immune escape by strongly inhibiting T-lymphocyte immunity [22]. The above results indicated that AIL blocked Treg-mediated immunosuppression and enhanced the cytotoxicity of T cells. To further verify the effect of AIL on Tregs, we isolated naive CD4 + T cells from the spleen of C57BL/6 mice. Additional file 2: Fig. S9B shows that the efficiency of naive CD4 + T-cell selection reached 97.3%. Then, naive CD4 + T cells were cultured with AIL-treated tumor supernatant under Treg-prone conditions for 3 days (Fig. 6 A). As shown in Fig. 6B-C, the percentage of Tregs was reduced following AIL-treated tumor supernatant treatment. Moreover, we further assessed the mRNA level of Foxp3 (a vital regulatory molecule expressed in Tregs that regulates the unique genetic features and transmission regulatory activity of Tregs [26]). In accordance with the flow cytometry results, AIL-treated tumor supernatant also inhibited the transcription of Foxp3 (Fig. 6D-E). Additionally, we assessed the effects of AIL on Treg differentiation (Additional file 2: Fig. S9A). AIL treatment also caused mild inhibition of Treg differentiation and Foxp3 mRNA levels (Additional file 2: Fig. S9C-E). A previous study showed that c-Jun was a TF of Foxp3 [37], indicating that the inhibitory effect of AIL on Tregs may be mediated by the AIL-c-Jun-Foxp3 axis. We then compared the effects of AIL and AIL-treated tumor supernatant on Treg differentiation. Interestingly, AIL-treated tumor supernatant exhibited more significant inhibitory effects on Treg differentiation and Foxp3 mRNA levels (Additional file 2: Fig. S9C-E), suggesting that the inhibitory effect of AIL on Treg differentiation is mainly determined by molecules in the tumor supernatant. It was previously reported that PD-L1 beads alone could convert naive CD4 + T cells to Foxp3 + Tregs in vitro [26]. We speculated that AIL might suppress the secretion of PD-L1 by targeting c-Jun, thus blocking Treg differentiation and further promoting the cytotoxicity of T cells. To confirm our hypothesis, we examined the effects of tumor supernatant on Treg differentiation after c-Jun overexpression in melanoma cells (Additional file 2: Fig. S9F). As expected, overexpression of c-Jun significantly promoted Treg differentiation (Additional file 2: Fig. S9G-H) and elevated Foxp3 mRNA levels (Additional file 2: Fig. S9I-J). In addition, overexpression of c-Jun partially rescued the Treg differentiation inhibition (Additional file 2: Fig. S9G-H) and Foxp3 downregulation (Additional file 2: Fig. 97I-J) caused by AIL. More importantly, our results showed that AIL-treated tumor supernatant inhibited Treg differentiation (Fig. 6 F-G) and Foxp3 expression (Fig. 6 H-I) more significantly after c-Jun overexpression, further suggesting that AIL indeed works by targeting c-Jun. Taken together, these results indicate that AIL inhibited Treg differentiation through the AIL-c-Jun-PD-L1 axis.

## Discussion

Compared with synthetic compounds, TCM monomers are extraordinarily valuable resources for the discovery of novel anticancer reagents [38–40], possessing higher biological activity and more diverse structures [41]. In this study, we performed a ligand-based screening workflow for identifying c-Jun inhibitors based on molecular docking combined with a consensus QSAR model from 1616 TCM monomers (Fig. 1A). After a series of selection criteria, AIL was manually screened as the candidate drug (Fig. 1A-C). We also predicted the targets of AIL by the combinatorial target prediction strategy (Additional file 2: Fig. S2A) and identified both c-Jun and AR with scores above 0.5 (Fig. 1D). Indeed, AIL was previously reported to induce AR degradation through the proteasome pathway [32], but AR scored lower than c-Jun, indicating the reliability of our screening model and c-Jun as the primary target for AIL. It has been reported that AIL can inhibit the progression of various tumors such as castration-resistant prostate cancer, non-small cell lung cancer and breast cancer [32, 42, 43]. In this study, we proved that AIL also inhibited melanoma progression and directly interacted with c-Jun and notably reduced its stability (Fig. 3), indicating that AIL is a promising therapeutic compound for melanoma treatment.

Anti-PD-1/PD-L1 treatment has provided substantial benefit for patients with advanced cancer. Various approaches are currently under way to improve the efficacy of these immune checkpoint inhibitors, such as combining immunotherapy with existing anticancer therapies, including chemotherapy, radiotherapy, or targeted therapies [44, 45]. Although these combinations have the potential to improve the outlook for cancer immunotherapy, they may also worsen immune-related adverse events with serious side effects [46]. Emerging evidence supports that TCM monomers contribute to tumor immunotherapy. Recently, low-dose Tubeimoside-1 (TBM-1) was found to significantly inhibit melanoma growth by inhibiting the expression of PD-L1 [47]. In addition, BBR is considered a negative regulator of PD-L1 and a promoter of antitumor immunity [48]. Here, we showed the synergistic effect of AIL and anti-PD-L1 mAb treatment on melanoma, reflected by the significant reduction in tumor volume and weight (Fig. 4 C and Additional file 2: Fig. S6A). Previous studies have shown that PD-L1 can be regulated by multiple key transcription factors, such as NF-κB, c-Myc, HIF-1α and STAT3 [49]. We also showed that c-Jun transcriptionally regulated PD-L1 expression by directly binding to the PD-L1 promoter region (Fig. 5G-J and Additional file 2: Fig. S7D).

**Fig. 7:**
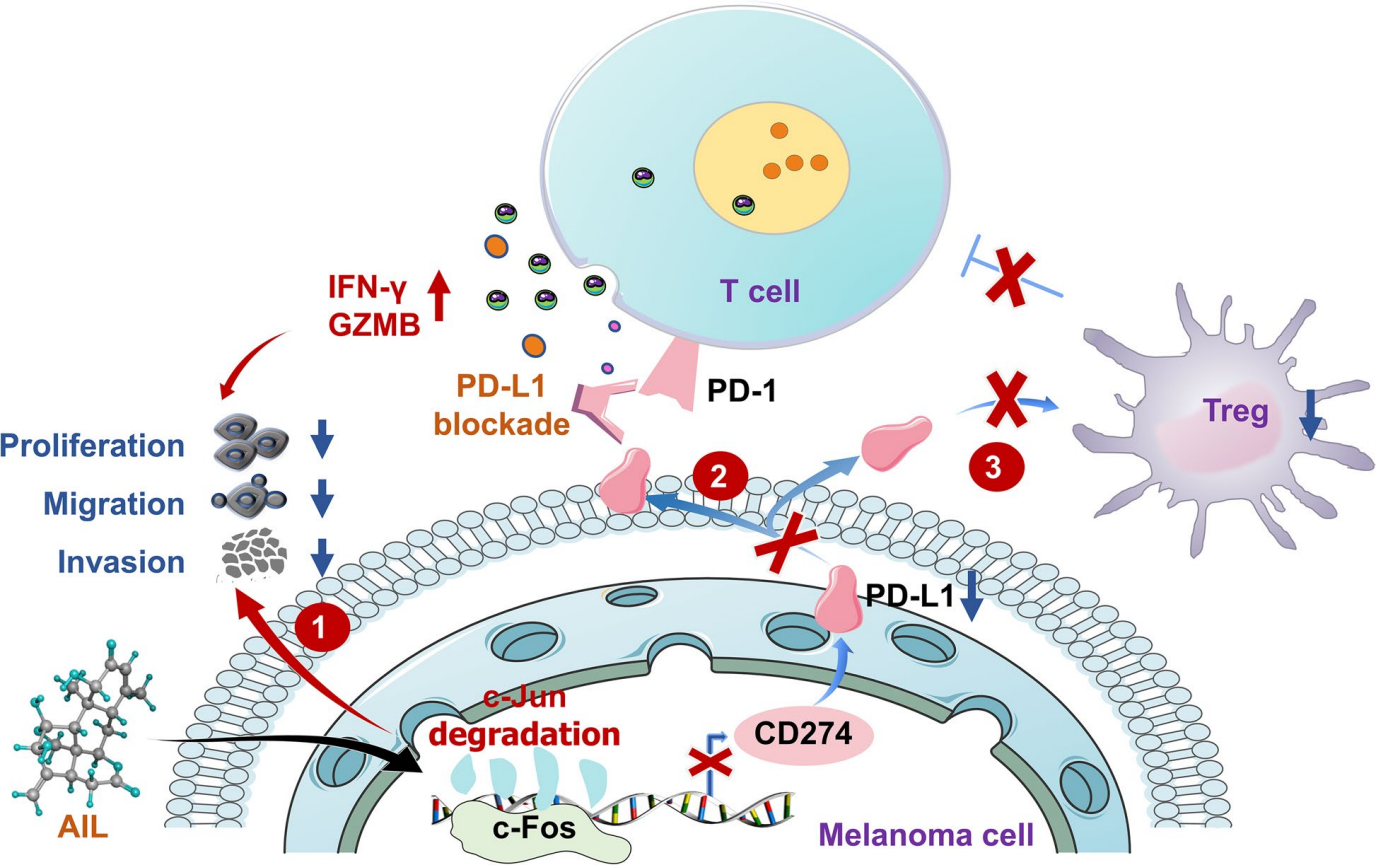
AIL enhances the effectiveness of anti-PD-L1 by promoting c-Jun degradation. AIL binds to and downregulates c-Jun by reducing its stability, thus suppressing melanoma progression. In addition, AIL suppresses PD-L1 transcription through targeting c-Jun in melanoma cells, consequently blocking PD-L1 secretion, resulting in Treg inhibition, and ultimately enhancing the efficacy of PD-L1 blockade

The high infiltration of Tregs is closely related to the progression of various types of cancer. Therefore, enhancing anti-tumor immunity by depleting Tregs and regulating Tregs function should be a promising strategy for tumor immunotherapy [50]. Intriguingly, our study revealed that the combination of AIL with anti-PD-L1 mAb facilitated the treatment efficacy of melanoma by suppressing Treg infiltration and enhancing CD8 + T cell activity (Fig. 4D-H). We constructed Treg differentiation model in vitro, and revealed extraordinary cooperation between melanoma cells and Tregs (Fig. 6 and Additional file 2: Fig. S9); that is, AIL inhibited PD-L1 secretion by targeting c-Jun in melanoma cells to suppress Treg differentiation, thus creating a highly efficient AIL-c-Jun-PD-L1-Treg antitumor pathway (Fig. 7).

## Conclusion

In summary, we screened the TCM monomer AIL as a novel c-Jun inhibitor that binds to and downregulates c-Jun by reducing its stability, thus suppressing melanoma progression. In addition, AIL suppressed PD-L1 transcription through the AIL-c-Jun-PD-L1 pathway in melanoma cells, consequently blocking PD-L1 secretion, resulting in Treg inhibition and Foxp3 expression reduction, and ultimately enhancing the efficacy of PD-L1 blockade. Therefore, our study suggests the potential value of AIL as a targeted inhibitor of c-Jun, either in isolation, or to enhance the effectiveness of the current PD-L1 checkpoint blockade immunotherapy in melanoma (Fig. 7).

## Supplementary Information

**Additional file 1:** **Table S1.** 114 TCM monomers. **Table S2.** The prediction performance of consensus SAR models basedon CATS, MACCS and MOE2D descriptors. **TableS3.** Predictive values of 25 hit compounds. **Table S4.** The ADMET properties of 6 hit compounds.

**Additional file 2:** **Fig. S1.** Screening of TCM monomer. (A) Chemical structures of the top 25 TCM monomer. **Fig. S2.** A virtual screening strategy identified c-Jun inhibitors. **Fig. S3.** AIL inhibited melanoma cell progression *in vitro.* **Fig. S4.** AIL directly interacted with c-Jun and promoted its degradation. **Fig. S5.** AIL directly interacted with c-Jun and promoted its degradation (quantification of Fig. 3). **Fig. S6.** Improved anti-tumor effect of anti-PD-L1 mAb and AIL cotreatment in B16-F10 tumor-bearing C57BL/6 mice. **Fig. S7.** AIL suppressed PD-L1 transcriptional expression through c-Jun. **Fig. S8.** AIL suppressed PD-L1 transcriptional expression through c-Jun (quantification of Fig. 5). **Fig. S9.** AIL blocked Treg differentiation through targeting c-Jun.

## Data Availability

The datasets during and/or analyzed during the current study are available from the corresponding author on reasonable request.

## Abbreviations

ACC: accuracy
ADMET: absorption, distribution, metabolism, excretion, and toxicity
AIL: *Ailanthone*
AP-1: Activator protein 1
ATCC: American Type Culture Collection
AUC: area under the curve
BRT: Britannin
bZIP: basic leucine zipper
CCK-8: Cell Counting Kit 8
ChIP: chromatin immunoprecipitation assay
CHX: cycloheximide
CST: Cell Signaling Technologies
DBD: DNA binding domain
DMEM: Dulbecco’s modified Eagle’s medium
DMEM/F-12: Dulbecco’s Modified Eagle Medium/Nutrient Mixture F-12
Foxp3: Forkhead box protein 3
FBS: fetal bovine serum
GZMB: granzyme B
ICI: immune checkpoint inhibitor
LZ: leucine zipper
MDSCs: myeloid-derived suppressor cells
MOE: Molecular Operating Environment
PD-1: programmed cell death protein 1
PD-L1: programmed death ligand-1
PVDF: polyvinylidene fluoride
QSAR: quantitative structure-activity relationship
SEA: Similarity Ensemble Approach
TCM: traditional Chinese medicine
TF: transcription factor
TME: tumor microenvironment
Tregs: regulatory T cells.
