# Supplementary material for: The traditional chinese medicine monomer Ailanthone improves the therapeutic efficacy of anti-PD-L1 in melanoma cells by targeting c-Jun
Source: J Exp Clin Cancer Res. 2022 Dec 15;41:346. doi: 10.1186/s13046-022-02559-z (PMC9753288; doi:10.1186/s13046-022-02559-z)
Supplement: Supplementary file 1 — Additional file 1: Table S1. 114 TCM monomers. Table S2. The prediction performance of consensus SAR models basedon CATS, MACCS and MOE2D descriptors. TableS3. Predictive values of 25 hit compounds. Table S4. The ADMET properties of 6 hit compounds. [file 13046_2022_2559_MOESM1_ESM.docx]

**Additional file 1: Table S1** 114 TCM monomers

| **Product Name** | **Product Name** | **Product Name** | **Product Name** | **Product Name** | **Product Name** |
| --- | --- | --- | --- | --- | --- |
| Betulinic acid | Cepharanthine | 18 β-Glycyrrhetinic acid | (E)-Ethyl p-methoxycinnamate | β-Elemonic acid | Pterostilbene |
| Chelerythrine (chloride) | Ayanin | Fisetin | Columbianadin | Hydroxytyrosol | Jaceosidin |
| Aloperine | Osthole | 4'-Hydroxychalcone | 18α-Glycyrrhetinic acid | Fraxetin | Tetrahydrocurcumin |
| Lupenone | Chlorogenic acid | Asiatic acid | Liquiritin | Terpinen-4-ol | Benzothiazole |
| Zingerone | Macelignan | 1,4-Cineole | Liquiritigenin | Morusin | Salvigenin |
| Sinomenine | Solasodine | Avicularin | Glabridin | Carnosic acid | Isorhapontigenin |
| Sinomenine hydrochloride | Sarsasapogenin | Fraxinellone | Decursinol | Aucubin | Methyl 3,4-dihydroxybenzoate |
| Resveratrol | Naringenin | Aloesin | Wogonin | Quercetagetin | Citric acid |
| Genipin | Isoliquiritigenin | Cordycepin | 4(3H)-Quinazolinone | trans-Chalcone | 4-Hydroxybenzyl alcohol |
| Decursin | Sophocarpine | Ligustrazine | Sophoricoside | Coumarin | Betulonic acid |
| Triptonide | Sophocarpine (monohydrate) | Caffeic acid phenethyl ester | Licochalcone D | Carvacrol | Ailanthone |
| Melatonin | Rhein | Colcemid | Acacetin | 8-O-Acetylharpagide | Artemisic acid |
| Sinapine | Salicin | Esculetin | (-)-Alkannin | Isoorientin | Withaferin A |
| (-)-Epicatechin | Nobiletin | Lycorine | Britannin | Isopimpinellin | Acetylshikonin |
| Oridonin | Oxymatrine | Lycorine (hydrochloride) | 6-Hydroxycoumarin | 1-beta-D-Arabinofuranosyluracil | Rhapontigenin |
| Baohuoside I | Luteolin | Ginsenoside Rk1 | Nitidine (chloride) | Isorhamnetin | Crebanine |
| Bergenin | Magnolol | Ziyuglycoside II | Gallic acid (hydrate) | Camphor | Curcumenol |
| Diphyllin | Beta-Sitosterol (purity>80%) | Farrerol | Allantoin | Abietic acid | Isoeugenol acetate |
| Arctigenin | Beta-Sitosterol (purity>98%) | Pseudolaric Acid B | Wedelolactone | Catalpol | Lapachol |

**Table S2** The prediction performance of consensus SAR models based on CATS, MACCS and MOE2D descriptors

|  | **Precision** | **Sensitivity** | **Specificity** | **Accuracy** | **AUC** |
| --- | --- | --- | --- | --- | --- |
| 1 | 0.777778 | 0.636364 | 0.826087 | 0.733333 | 0.795455 |
| 2 | 0.590909 | 0.590909 | 0.608696 | 0.6 | 0.703557 |
| 3 | 0.727273 | 0.727273 | 0.73913 | 0.733333 | 0.822134 |
| 4 | 0.888889 | 0.727273 | 0.913043 | 0.822222 | 0.865613 |
| 5 | 0.894737 | 0.772727 | 0.913043 | 0.844444 | 0.909091 |
| 6 | 0.8125 | 0.590909 | 0.869565 | 0.733333 | 0.843874 |
| 7 | 0.703704 | 0.863636 | 0.652174 | 0.755556 | 0.86166 |
| 8 | 0.666667 | 0.727273 | 0.652174 | 0.688889 | 0.794466 |
| 9 | 0.695652 | 0.727273 | 0.695652 | 0.711111 | 0.757905 |
| 10 | 0.789474 | 0.681818 | 0.826087 | 0.755556 | 0.871542 |
| 11 | 0.791667 | 0.863636 | 0.782609 | 0.822222 | 0.920949 |
| 12 | 0.692308 | 0.818182 | 0.652174 | 0.733333 | 0.882411 |
| 13 | 0.818182 | 0.818182 | 0.826087 | 0.822222 | 0.854743 |
| 14 | 0.681818 | 0.681818 | 0.695652 | 0.688889 | 0.826087 |
| 15 | 0.76 | 0.863636 | 0.73913 | 0.8 | 0.911067 |
| 16 | 0.666667 | 0.636364 | 0.695652 | 0.666667 | 0.816206 |
| 17 | 0.75 | 0.818182 | 0.73913 | 0.777778 | 0.875494 |
| 18 | 0.708333 | 0.772727 | 0.695652 | 0.733333 | 0.804348 |
| 19 | 0.75 | 0.954545 | 0.695652 | 0.822222 | 0.889328 |
| 20 | 0.733333 | 0.5 | 0.826087 | 0.666667 | 0.767787 |
| average | 0.744994 | 0.738636 | 0.752174 | 0.745556 | 0.838686 |

**Table S3** Predictive values of 25 hit compounds

| **Product Name** | **P** | **P** | **P** | **P** | **P** | **P** | **P** | **P** | **P** | **P** | **P_ave** |
| --- | --- | --- | --- | --- | --- | --- | --- | --- | --- | --- | --- |
| Withaferin A | 0.644 | 0.6 | 0.602 | 0.612 | 0.642 | 0.588 | 0.628 | 0.624 | 0.598 | 0.61 | 0.6148 |
| Britannin | 0.582 | 0.592 | 0.574 | 0.582 | 0.604 | 0.594 | 0.592 | 0.576 | 0.578 | 0.58 | 0.5854 |
| Ailanthone | 0.59 | 0.578 | 0.552 | 0.56 | 0.57 | 0.58 | 0.572 | 0.622 | 0.578 | 0.58 | 0.5782 |
| Columbianadin | 0.524 | 0.552 | 0.532 | 0.554 | 0.548 | 0.546 | 0.56 | 0.564 | 0.572 | 0.56 | 0.5512 |
| Macelignan | 0.49 | 0.486 | 0.528 | 0.512 | 0.508 | 0.486 | 0.532 | 0.516 | 0.508 | 0.532 | 0.5098 |
| Nobiletin | 0.514 | 0.522 | 0.498 | 0.462 | 0.506 | 0.506 | 0.502 | 0.48 | 0.51 | 0.5 | 0.5 |
| Liquidity | 0.248 | 0.258 | 0.246 | 0.256 | 0.232 | 0.264 | 0.29 | 0.254 | 0.258 | 0.286 | 0.2592 |
| Tetrahydrocurcumin | 0.188 | 0.19 | 0.198 | 0.188 | 0.214 | 0.208 | 0.192 | 0.186 | 0.204 | 0.208 | 0.1976 |
| Sophoricoside | 0.224 | 0.21 | 0.208 | 0.212 | 0.21 | 0.198 | 0.216 | 0.202 | 0.23 | 0.226 | 0.2136 |
| Morusin | 0.226 | 0.292 | 0.202 | 0.228 | 0.236 | 0.244 | 0.222 | 0.242 | 0.234 | 0.22 | 0.2346 |
| Arctigenin | 0.474 | 0.492 | 0.488 | 0.468 | 0.474 | 0.492 | 0.512 | 0.502 | 0.518 | 0.434 | 0.4854 |
| Ziyuglycoside II | 0.248 | 0.244 | 0.23 | 0.272 | 0.23 | 0.244 | 0.238 | 0.232 | 0.24 | 0.208 | 0.2386 |
| Pseudolaric Acid B | 0.42 | 0.476 | 0.398 | 0.402 | 0.426 | 0.41 | 0.42 | 0.418 | 0.418 | 0.372 | 0.416 |
| Sinapine | 0.286 | 0.278 | 0.278 | 0.286 | 0.31 | 0.284 | 0.276 | 0.296 | 0.274 | 0.302 | 0.287 |
| Licochalcone D | 0.308 | 0.384 | 0.318 | 0.298 | 0.308 | 0.318 | 0.286 | 0.326 | 0.358 | 0.324 | 0.3228 |
| Baohuoside I | 0.264 | 0.286 | 0.24 | 0.252 | 0.23 | 0.248 | 0.264 | 0.266 | 0.276 | 0.244 | 0.257 |
| Avicularin | 0.216 | 0.2 | 0.18 | 0.182 | 0.208 | 0.176 | 0.184 | 0.198 | 0.204 | 0.2 | 0.1948 |
| 18β-Glycyrrhetinic acid | 0.4 | 0.34 | 0.328 | 0.388 | 0.362 | 0.348 | 0.346 | 0.336 | 0.392 | 0.322 | 0.3562 |
| Betulonic acid | 0.422 | 0.382 | 0.364 | 0.398 | 0.374 | 0.352 | 0.376 | 0.336 | 0.394 | 0.36 | 0.3758 |
| 8-O-Acetylharpagide | 0.228 | 0.306 | 0.258 | 0.266 | 0.23 | 0.264 | 0.28 | 0.212 | 0.242 | 0.256 | 0.2542 |
| Asiatic acid | 0.398 | 0.336 | 0.304 | 0.376 | 0.336 | 0.33 | 0.342 | 0.304 | 0.362 | 0.326 | 0.3414 |
| Cepharanthine | 0.37 | 0.34 | 0.324 | 0.302 | 0.302 | 0.328 | 0.352 | 0.33 | 0.322 | 0.304 | 0.3274 |
| Magnolol | 0.35 | 0.344 | 0.354 | 0.34 | 0.398 | 0.35 | 0.326 | 0.362 | 0.33 | 0.366 | 0.352 |
| Chlorogenic acid | 0.156 | 0.162 | 0.152 | 0.174 | 0.15 | 0.158 | 0.176 | 0.154 | 0.16 | 0.12 | 0.1562 |
| Betulinic acid | 0.416 | 0.362 | 0.342 | 0.406 | 0.372 | 0.338 | 0.37 | 0.348 | 0.394 | 0.354 | 0.3702 |

**Table S4** The ADMET properties of 6 hit compounds

| **Product Name** | **logP** | **HIA** | **F** | **BBB** | **T (1/2)** | **hERG** | **H-HT** | **Ames** |
| --- | --- | --- | --- | --- | --- | --- | --- | --- |
| Macelignan | 4.187 | 0.633 | 0.462 | 0.885 | 1.738 | 0.688 | 0.688 | 0.42 |
| Withaferin A | 3.353 | 0.554 | 0.425 | 0.892 | 1.709 | 0.477 | 0.477 | 0.198 |
| Nobiletin | 3.512 | 0.699 | 0.557 | 0.927 | 1.941 | 0.723 | 0.723 | 0.35 |
| Britannin | 1.374 | 0.591 | 0.317 | 0.893 | 1.045 | 0.185 | 0.185 | 0.322 |
| Columbianadin | 3.385 | 0.647 | 0.466 | 0.885 | 1.511 | 0.49 | 0.49 | 0.524 |
| Ailanthone | 0.086 | 0.472 | 0.445 | 0.826 | 0.919 | 0.224 | 0.224 | 0.444 |
