# Supplementary material for: The traditional chinese medicine monomer Ailanthone improves the therapeutic efficacy of anti-PD-L1 in melanoma cells by targeting c-Jun
Source: J Exp Clin Cancer Res. 2022 Dec 15;41:346. doi: 10.1186/s13046-022-02559-z (PMC9753288; doi:10.1186/s13046-022-02559-z)
Supplement: Supplementary file 2 — Additional file 2: Fig. S1. Screening of TCM monomer. (A) Chemical structures of the top 25 TCM monomer. Fig. S2. A virtual screening strategy identified c-Jun inhibitors. Fig. S3. AIL inhibited melanoma cell progression in vitro. Fig. S4. AIL directly interacted with c-Jun and promoted its degradation. Fig. S5. AIL directly interacted with c-Jun and promoted its degradation (quantification of Fig. 3). Fig. S6. Improved anti-tumor effect of anti-PD-L1 mAb and AIL cotreatment in B16-F10 tumor-bearing C57BL/6 mice. Fig. S7. AIL suppressed PD-L1 transcriptional expression through c-Jun. Fig. S8. AIL suppressed PD-L1 transcriptional expression through c-Jun (quantification of Fig. 5). Fig. S9. AIL blocked Treg differentiation through targeting c-Jun. [file 13046_2022_2559_MOESM2_ESM.docx]

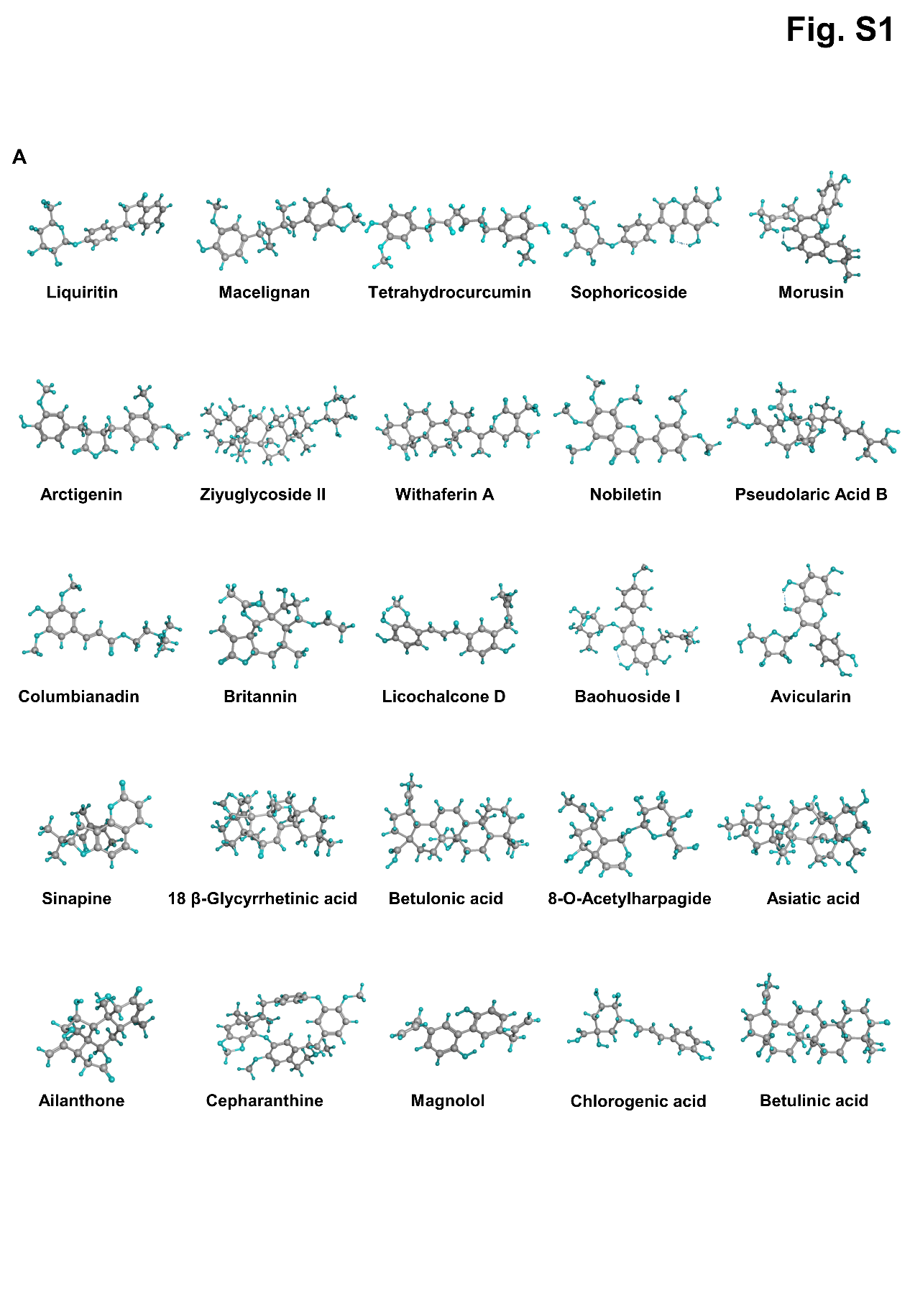


**Fig. S1.** Screening of TCM monomer. (A) Chemical structures of the top 25 TCM monomer.


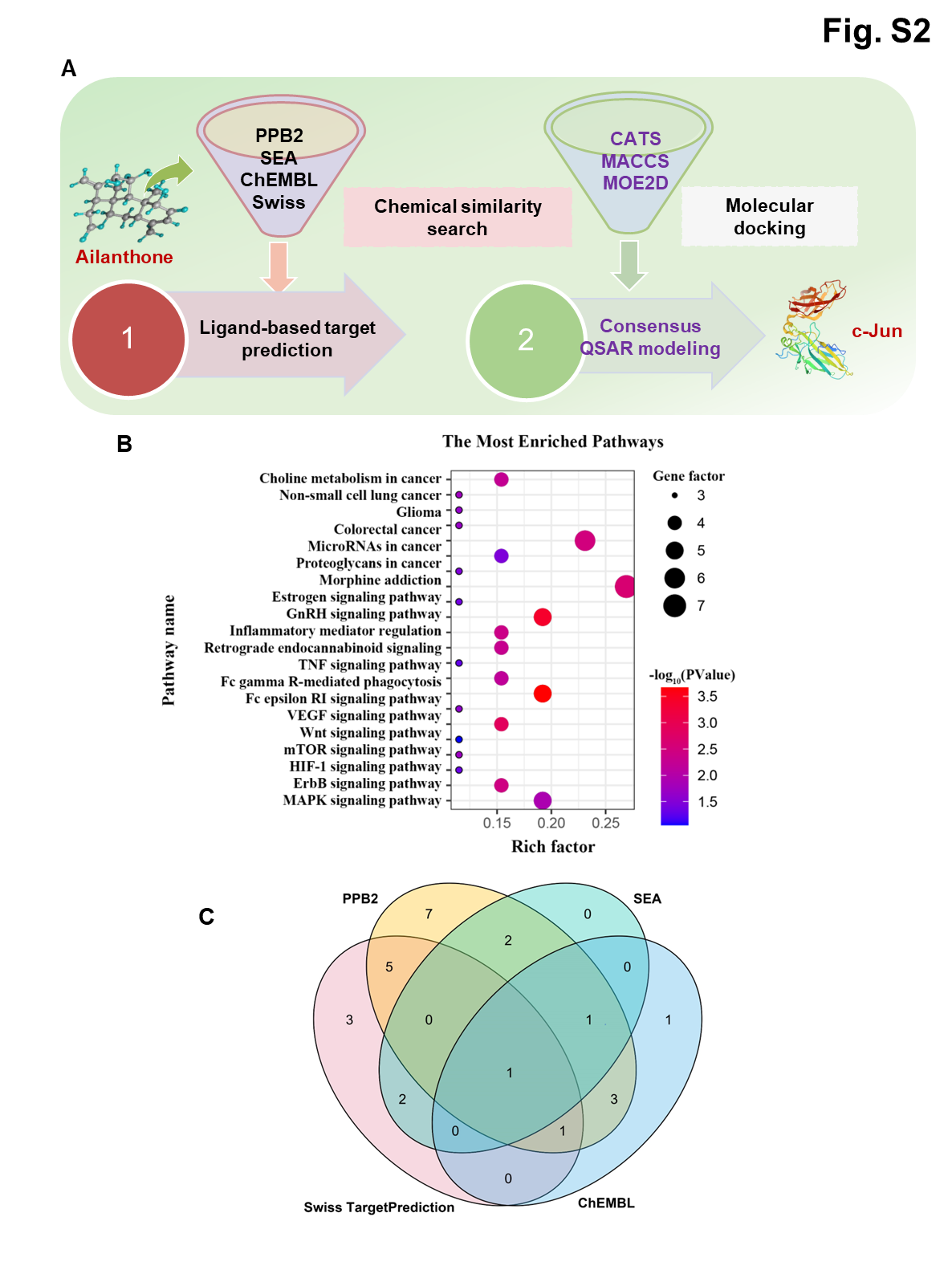


**Fig. S2.** A virtual screening strategy identified c-Jun inhibitors. (A) A combinatorial target prediction strategy to screen the potential targets of AIL stepwise. (B) KEGG pathway analysis to validate the effects of targets. (C) Overlapping targets of AIL predicted by diverse target prediction tools.


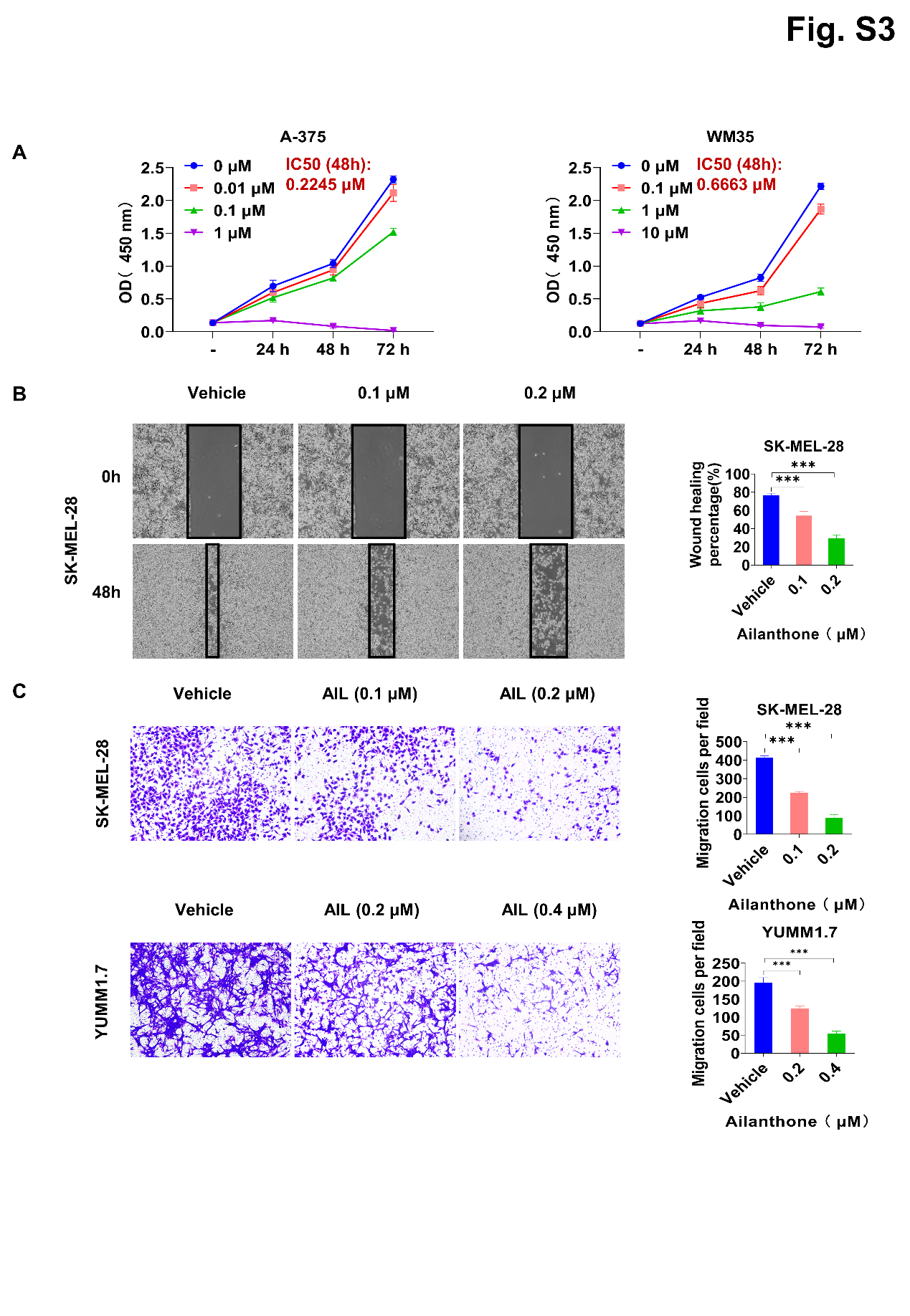


**Fig. S3.** AIL inhibited melanoma cell progression *in vitro.* (A) A-375 and WM35 cells were treated with the indicated concentrations of AIL for 0, 24, 48 and 72 h. Cell viability was determined with the CCK-8 reagent. The average IC_50_ values for AIL in A-375 and WM35 are shown. (B) Wound healing assay in SK-MEL-28 and YUMM1.7 cells treated with AIL. (C) Transwell invasion assay in SK-MEL-28 cells treated with AIL. Data were shown as the mean ± SEM of three independent experiments. Data are shown as the mean ± SEM, n = 3; **p* < 0.05, ***p* < 0.01 and ****p* < 0.001.


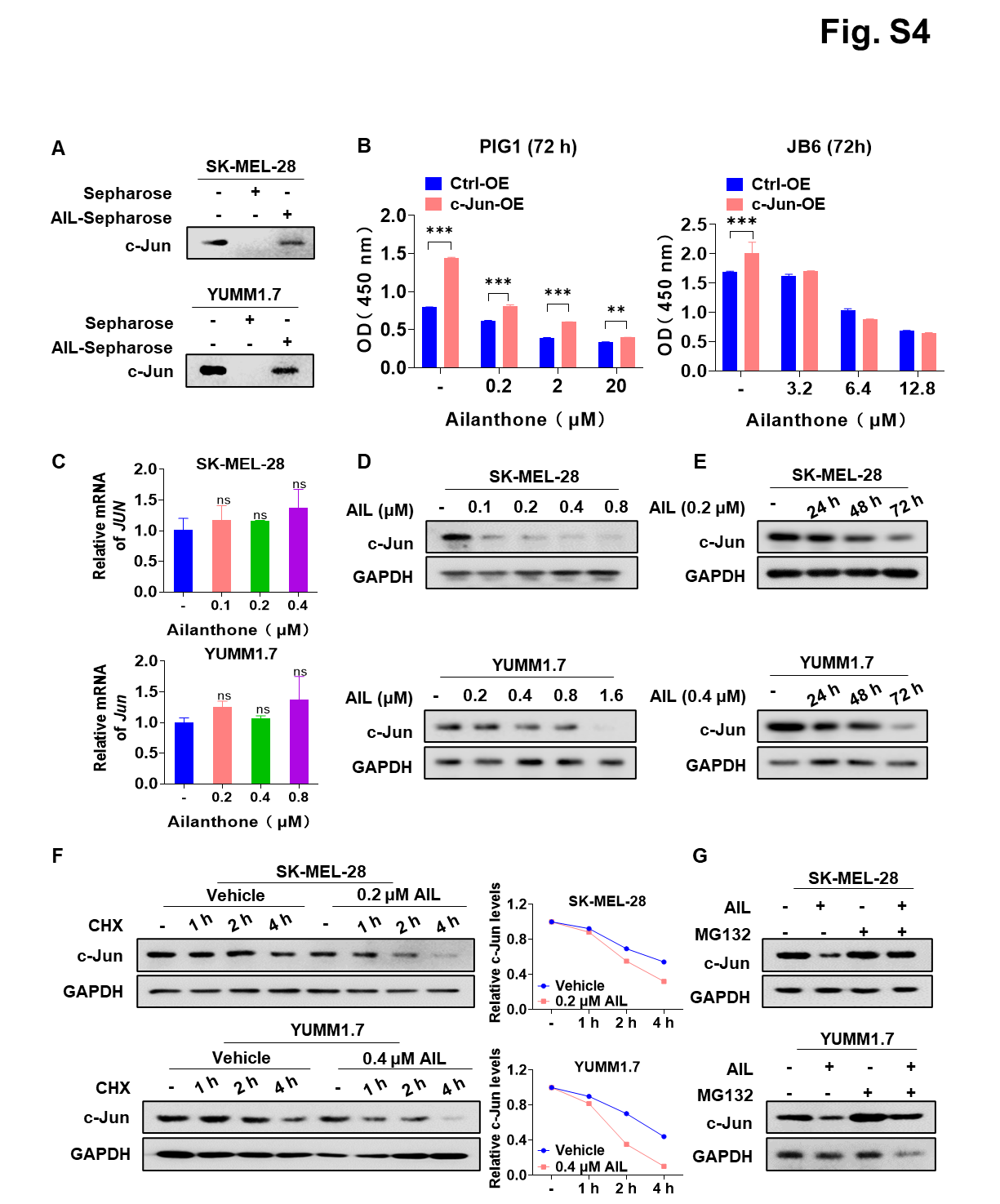


**Fig. S4.** AIL directly interacted with c-Jun and promoted its degradation. (A) SK-MEL-28 and YUMM1.7 cell lysates were detected by western blot with anti-c-Jun antibody for pull-down assay. (B) PIG1 and JB6 cells were transfected with c-Jun overexpression or negative control vector, and then treated with various concentrations of AIL for 72 h. The cell viability was examined by CCK-8 assay. (C) SK-MEL-28 and YUMM1.7 cells were treated with the indicated concentrations of AIL for 24 h. QRT-PCR was performed to analyze c-Jun mRNA levels. (D) SK-MEL-28 and YUMM1.7 cells were treated with the indicated concentrations of AIL for 24 h. Western blot was applied to detect c-Jun expression. GAPDH was used as a loading control. (E) SK-MEL-28 and YUMM1.7 cells were treated with AIL for different lengths of time. Western blot was applied to detect the c-Jun protein level. GAPDH was used as a loading control. (F) SK-MEL-28 and YUMM1.7 cells were treated with cycloheximide (CHX, 200 μg/mL) with or without AIL for the indicated time. The c-Jun protein level was measured by western blot. GAPDH was used as a loading control. (G) SK-MEL-28 and YUMM1.7 cells were treated with AIL with or without MG-132 (30 μmol/L), and c-Jun protein levels were measured by western blot. GAPDH was used as a loading control. Data are shown as the mean ± SD, n = 3; *ns*, not significant; **p* < 0.05, ***p* < 0.01 and ****p* < 0.001.


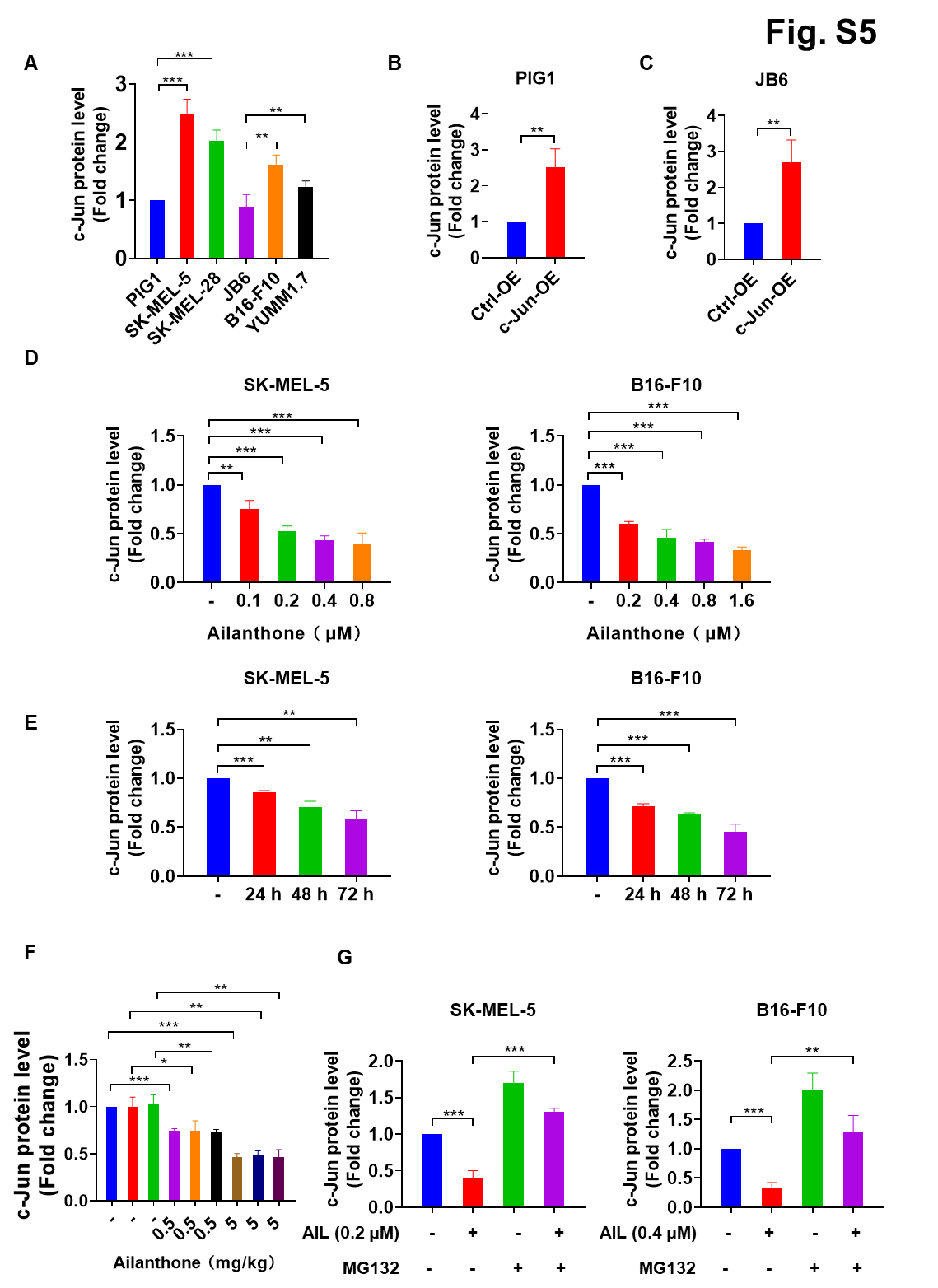


**Fig. S5.** AIL directly interacted with c-Jun and promoted its degradation (quantification of Fig. 3). (A) Differential expression levels of c-Jun in PIG1, SK-MEL-5, SK-MEL-28, JB6, B16-F10 and YUMM1.7 cells. The c-Jun protein level in PIG1, SK-MEL-5, SK-MEL-28, JB6, B16-F10 and YUMM1.7 cells was measured by western blot. GAPDH was used as the loading control. Quantifications of c-Jun to GAPDH were shown. (B-C) PIG1 and JB6 cells were transfected with c-Jun overexpression or negative control vector, and c-Jun expression was analyzed by western blot. GAPDH served as the loading control. Quantifications of c-Jun to GAPDH were shown. (D) SK-MEL-5 and B16-F10 cells were treated with the indicated concentrations of AIL for 24 h. Western blot was applied to detect c-Jun expression. GAPDH was used as a loading control. Quantifications of c-Jun to GAPDH were shown. (E) SK-MEL-5 and B16-F10 cells were treated with AIL for different lengths of time. Western blot was applied to detect the c-Jun protein level. GAPDH was used as a loading control. Quantifications of c-Jun to GAPDH were shown. (F) Western blot analysis of the expression of c-Jun in tumor samples with the indicated concentrations of AIL treatment. GAPDH was used as a loading control. Quantifications of c-Jun to GAPDH were shown. (G) SK-MEL-5 and B16-F10 cells were treated with AIL with or without MG-132 (30 μmol/L), and c-Jun protein levels were measured by western blot. GAPDH was used as a loading control. Quantifications of c-Jun to GAPDH were shown. Data are shown as the mean ± SEM, n = 3; *ns*, not significant; **p* < 0.05, ***p* < 0.01 and ****p* < 0.001.


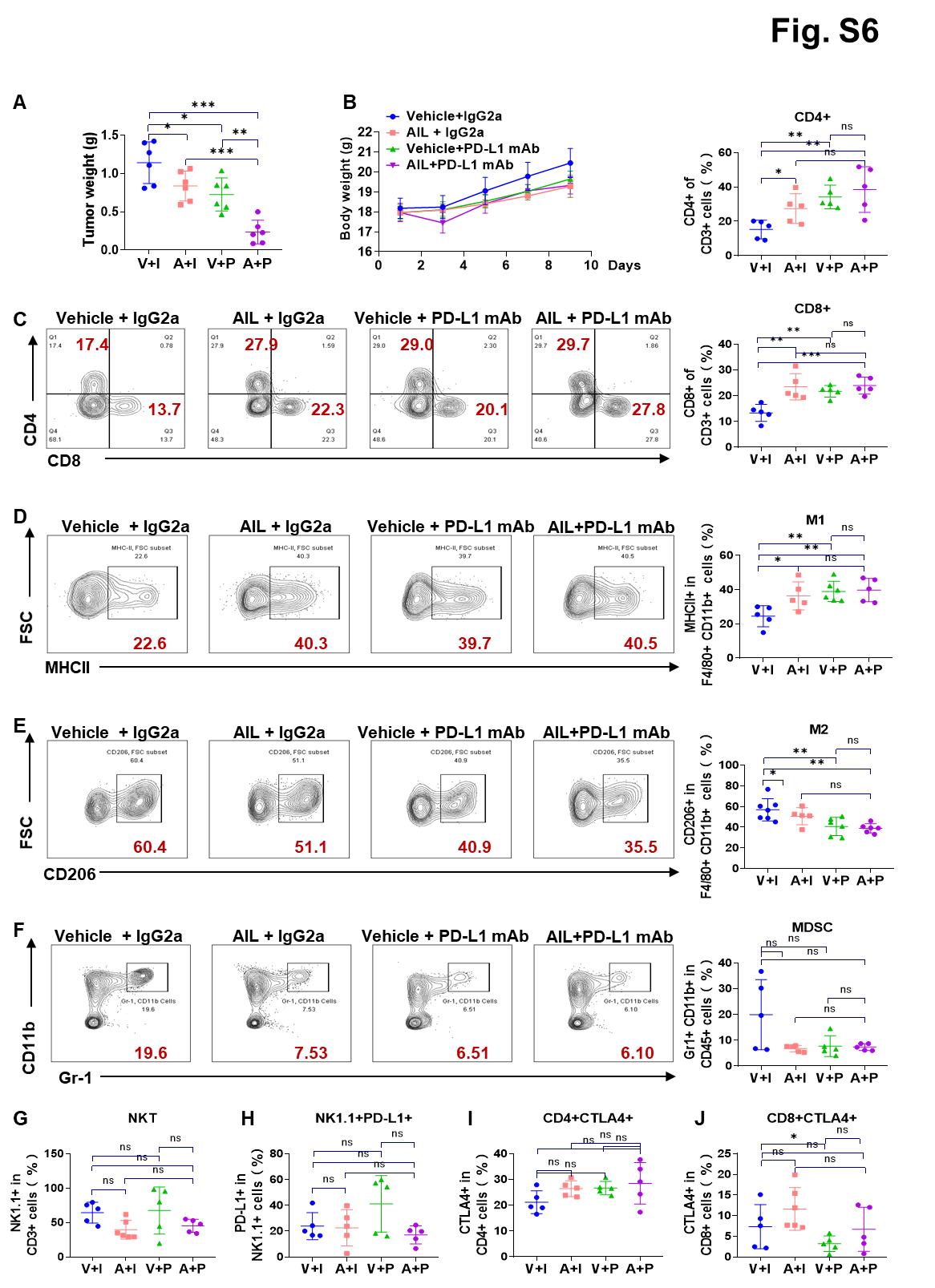


**Fig. S6.** Improved anti-tumor effect of anti-PD-L1 mAb and AIL cotreatment in B16-F10 tumor-bearing C57BL/6 mice. (A-B) C57BL/6 mice were implanted with 5 × 10^5^ B16-F10 cells and received PBS, AIL, IgG isotype control (IgG2a) or PD-L1 mAb treatment. (A) Tumor weight. (B) The body weight curves of the mice measured every two days. (C) Representative flow cytometric plots and quantification of CD4+ and CD8^+^ in CD3+ cells after the indicated treatments. (D) Representative flow cytometric plots and quantification of MHCII+ in F4/80+CD11b+ cells after the indicated treatments. (E) Representative flow cytometric plots and quantification of CD206+ in F4/80+CD11b+ cells after the indicated treatments. (F) Representative flow cytometric plots and quantification of Gr-1+ CD11b+ in CD45+ cells after the indicated treatments. (G) Quantification of NK1.1 in CD3+ cells. (H) Quantification of PD-L1+ in NK1.1+ cells. (I) Quantification of CTLA4+ in CD4+ cells. (J) Quantification of CTLA4+ in CD8+ cells. Data were shown as mean ± SEM, n = 5; *ns*, not significant; **p* < 0.05, ***p* < 0.01 and ****p* < 0.001.


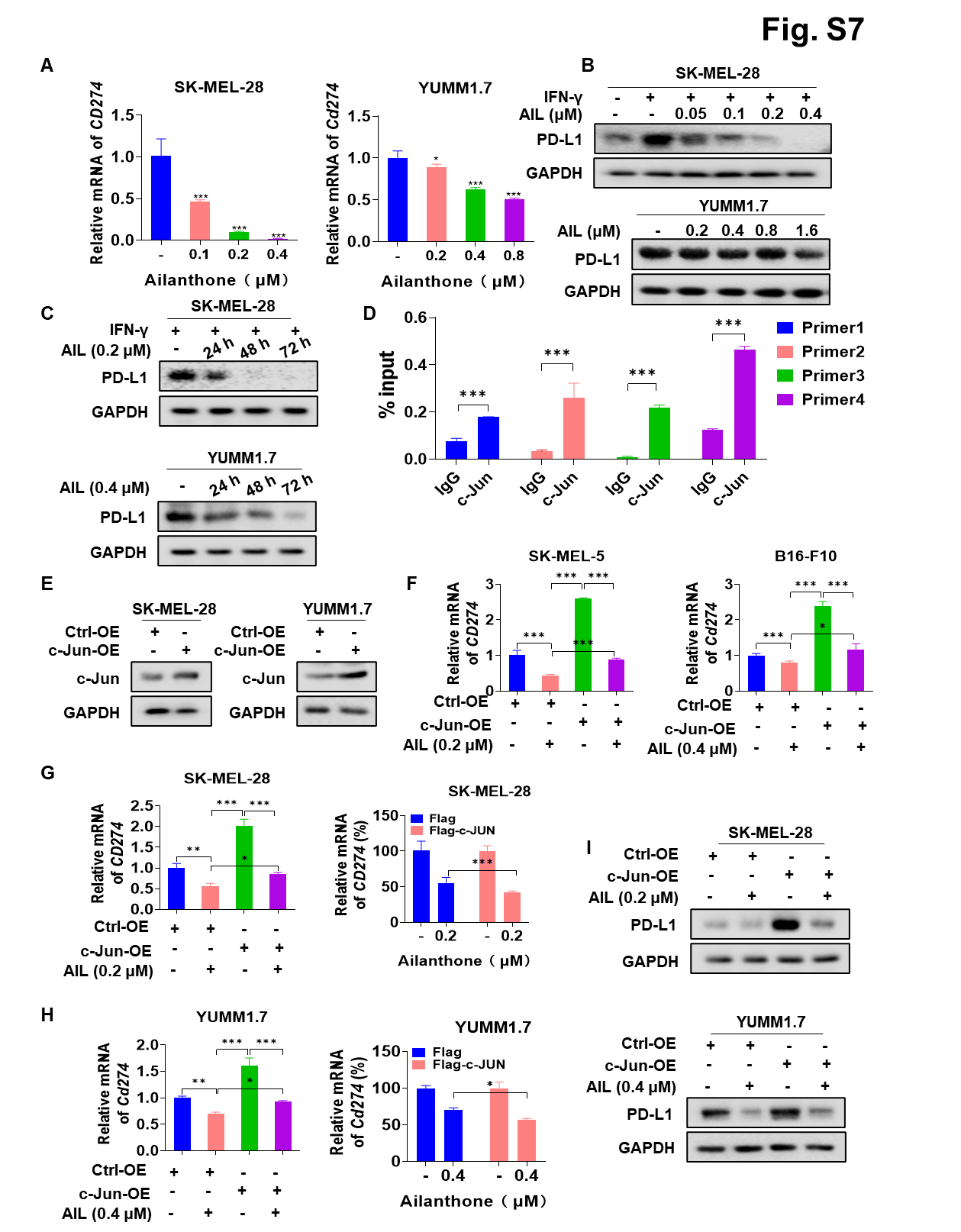


**Fig. S7.** AIL suppressed PD-L1 transcriptional expression through c-Jun. (A) SK-MEL-28 and YUMM1.7 cells were treated with AIL indicated concentration for 24 h. QRT-PCR was performed to analyze PD-L1 mRNA level. (B) SK-MEL-28 and YUMM1.7 cells were treated with the indicated concentrations of AIL for 24 h. Western blot was applied to detect PD-L1 expression. GAPDH was used as a loading control. (C) SK-MEL-28 and YUMM1.7 cells were treated with AIL for different lengths of time. Western blot was applied to detect the PD-L1 protein level. GAPDH was used as a loading control. (D) ChIP-qPCR assays for c-Jun binding to the PD-L1 promoter sites in SK-MEL-28 cells. (E) The transfection effect of the c-Jun overexpression plasmid was verified by western blot in SK-MEL-28 and YUMM1.7 cells. (F) PD-L1 mRNA level in SK-MEL-5 and B16-F10 cells with overexpressing vector or c-Jun upon AIL treatment were analyzed by qRT–PCR. (G) PD-L1 mRNA level and the relative PD-L1 mRNA level in SK-MEL-28 cells overexpressing vector or c-Jun upon AIL treatment were analyzed by qRT–PCR. (H) PD-L1 mRNA level and the relative PD-L1 mRNA level in YUMM1.7 cells overexpressing vector or c-Jun upon AIL treatment were analyzed by qRT–PCR. (I) PD-L1 expression in SK-MEL-28 and YUMM1.7 cells overexpressing vector or c-Jun upon AIL treatment was detected by western blot. Data were shown as mean ± SD, n = 3; **p* < 0.05, ***p* < 0.01 and ****p* < 0.001.


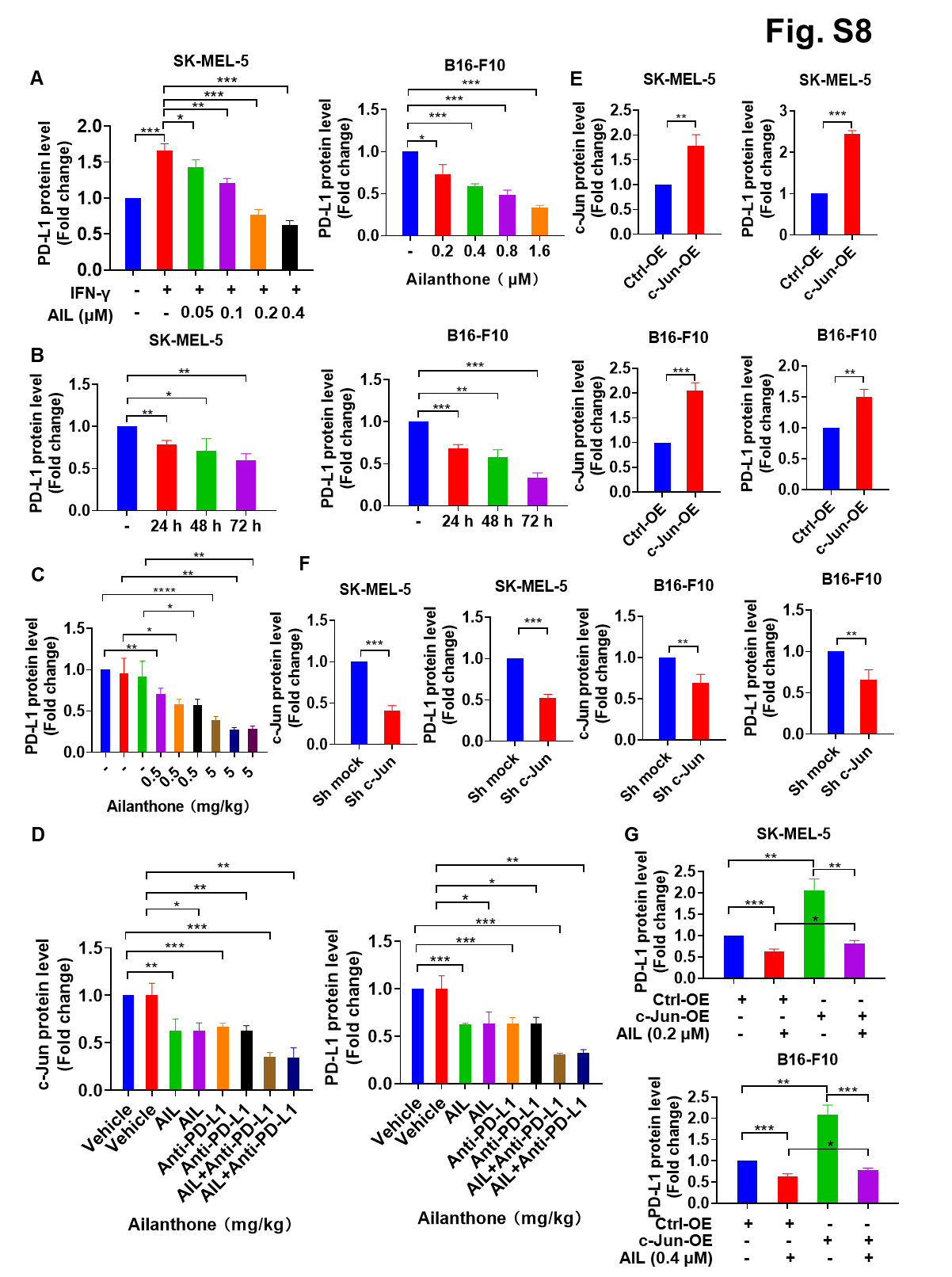


**Fig. S8.** AIL suppressed PD-L1 transcriptional expression through c-Jun (quantification of Fig. 5). (A) SK-MEL-5 and B16-F10 cells were treated with the indicated concentrations of AIL for 24 h. Western blot was applied to detect PD-L1 expression. GAPDH was used as a loading control. Quantifications of PD-L1 to GAPDH were shown. (B) SK-MEL-5 and B16-F10 cells were treated with AIL for different lengths of time. Western blot was applied to detect the PD-L1 protein level. GAPDH was used as a loading control. Quantifications of PD-L1 to GAPDH were shown. (C) Western blot analysis of the expression of PD-L1 in tumor samples with the indicated concentrations of AIL treatment. GAPDH was used as a loading control. Quantifications of PD-L1 to GAPDH were shown. (D) Western blot analysis of the expression of c-Jun and PD-L1 in tumor samples with anti-PD-L1 mAb and AIL cotreatment. GAPDH was used as a loading control. Quantifications of c-Jun and PD-L1 to GAPDH were shown. (E) c-Jun and PD-L1 expression in SK-MEL-5 and B16-F10 cells overexpressing vector or c-Jun was detected by western blot in SK-MEL-5 and B16-F10 cells. Quantifications of c-Jun and PD-L1 to GAPDH were shown. (F) c-Jun and PD-L1 expression in SK-MEL-5 and B16-F10 cells with c-Jun knockdown was detected by western blot. Quantifications of c-Jun and PD-L1 to GAPDH were shown. (G) PD-L1 expression in SK-MEL-5 and B16-F10 cells overexpressing vector or c-Jun upon AIL treatment was detected by western blot. Quantifications of PD-L1 to GAPDH were shown. Data are shown as the mean ± SEM, n = 3; **p* < 0.05, ***p* < 0.01 and ****p* < 0.001.

**
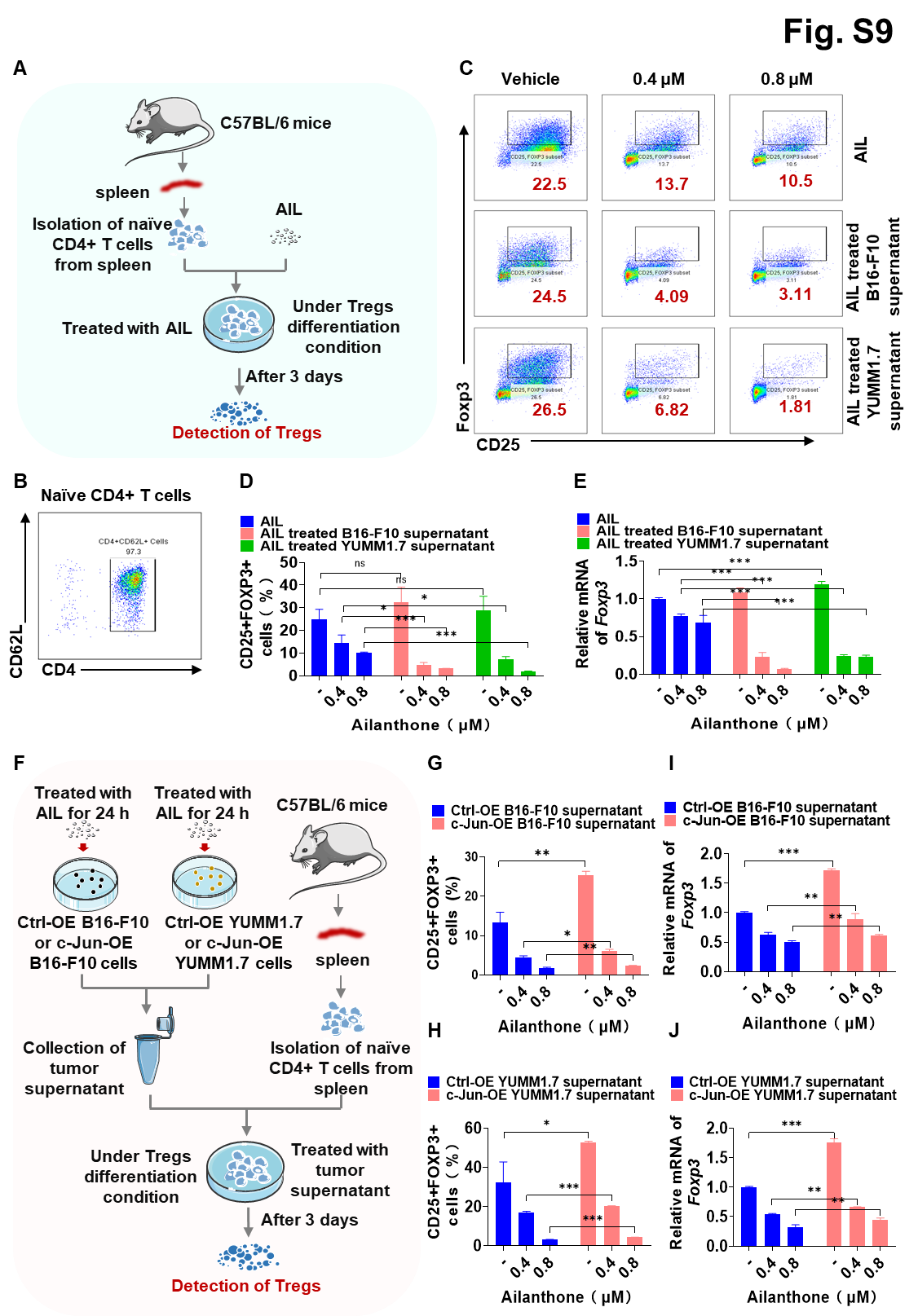
**

**Fig. S9.** AIL blocked Treg differentiation through targeting c-Jun. (A) Naïve CD4+ T cells were isolated from the spleen of C57BL/6 mice, seeded at a density of 1 × 10^6^ cells per well in 24-well plates, and finally cultured with AIL under Treg-prone conditions. After 3 days, CD25+Foxp3+ cells were detected by flow cytometry and Foxp3 mRNA levels were determined by qRT-PCR. (B) The efficiency of CD4+ cells selection was measured by flow cytometry. (C) Representative flow cytometric plots of CD25+Foxp3+ in CD4+ cells after the indicated treatments. (D) Relative quantification of Tregs differentiated from naive CD4+ T cells. (E) Foxp3 mRNA level. (F-J) Naive CD4+ T cells were isolated from the spleen of C57BL/6 mice, seeded at a density of 1 × 10^6^ cells per well in 24-well plates, and finally cultured with AIL-treated vector/c-Jun overexpressing B16-F10 supernatant or AIL-treated vector/c-Jun overexpressed YUMM1.7 supernatant under Treg-prone conditions. After 3 days, CD25+Foxp3+ cells were detected by flow cytometry and Foxp3 mRNA levels were determined by qRT-PCR. (F) Schematic of the treatment plan. (G-H) Representative flow cytometric plots and relative quantification of CD25+Foxp3+ in CD4+ cells after the indicated treatments. (I-J) Foxp3 mRNA level. Data were shown as mean ± SD, n = 3; **p* < 0.05, ***p* < 0.01 and ****p* < 0.001.
